# Nutrient intake, introduction of baby cereals and other complementary foods in the diets of infants and toddlers from birth to 23 months of age

**DOI:** 10.3934/publichealth.2020012

**Published:** 2020-03-04

**Authors:** Theresa A Nicklas, Carol E O'Neil, Victor L Fulgoni

**Affiliations:** 1 USDA/ARS Children's Nutrition Research Center, Baylor College of Medicine, Houston, 77030, USA; 2 Louisiana State University Agricultural Center (Emeritus), 143 Kenilworth Parkway, Baton Rouge, LA 70808, USA; 3 Nutrition Impact, LLC, 9725 Drive North, Battle Creek, MI, 49014, USA

**Keywords:** NHANES, infants, toddlers, baby cereal, baby rice cereal, complementary foods

## Abstract

**Introduction:**

Understanding which foods are introduced in the diet and when provides valuable information on complementary feeding. Fortified baby rice cereal is the most common first solid food given to infants, often followed by other baby cereals. The objective of this study was to examine food patterns among infants and toddlers consuming rice or non-rice baby cereals versus non-consumers.

**Methods:**

NHANES 2001–2014 data were used to assess dietary intake, nutrient adequacy, and food specific patterns of infants and toddlers. Groups were: baby cereal non-consumers (n = 3,910), non-rice baby cereal consumers (n = 711), and rice baby cereal consumers (n = 966). Those consuming both non-rice cereal and rice cereal were included in the rice cereal group (n = 9, 48, 61, and 10 for those 0–3, 4–6, 7–11, and 12–23 mos, respectively). Least-square means ± SEs were determined for nutrient intake and food group consumption by using covariate controlled regression analyses (p < 0.01).

**Results:**

Baby cereal consumer groups, compared to non-consumers, had higher intakes of carbohydrates, iron, calcium, magnesium, zinc, and vitamin E, with lower percentage having intakes below the Estimated Average Requirement for iron, calcium, and vitamin E. Infants 0–3 mos and 4–6 mos in both baby cereal consumption groups consumed other solid foods, including baby foods and beverages, sweetened beverages, coffee and tea, 100% juice, vegetables (excluding potatoes), fruit, sugars, milk and yogurt, and mixed dishes. The baby cereal consumers and non-consumers groups had intakes aligned with the “American diet”. Baby cereal non-consumers had a significantly higher percentage of exclusively breast fed at ages 0–3 mos and a lower percentage formula fed.

**Conclusion:**

This study provides detailed information on the introduction of baby cereals which was associated with better nutrient intakes and other complementary foods and intakes of nutrients that require special attention during early life. Further, cow's milk products and solid foods were introduced prior to the American Academy of Pediatrics' age recommendations.

## 1. Introduction

Understanding the dietary intakes of infants and toddlers is important because early life nutrition influences future health outcomes [1]. Although authoritative recommendations exist regarding the feeding of breast milk, infant formula, and cow's milk [2]–[4], the guidelines for feeding solid foods to children are to introduce solid foods between 4 to 6 months of age (mos) [5], depending on physiologic and developmental maturation [5],[6]. The Agricultural Act of 2014 (known as the Farm Bill) directed the Dietary Guidelines for Americans (DGA) to expand their mandate by including development of recommendations for infants from birth to 24 mos beginning with the 2020 DGA (the B-24 Project) [7],[8]. Data collected through the National Health and Nutrition Examination Survey (NHANES) have many potential uses in this regard. The new mandate also called for identification of key research questions and data gaps as the government moves forward with the 2020 DGA [9]. Thus, for the first time, recommendations for helping to provide dietary guidance such that the path to healthy eating starts as early in life as possible.

Dietary intake in infants and toddlers is understudied. Existing data suggest that, for infants and toddlers, the micronutrients that are most likely to be low or deficient (depending on the developmental age and the time and amount of introducing complementary foods and table foods) are dietary fiber, iron; calcium; zinc; magnesium; potassium; vitamins B_6_, D, E, and A, along with high intakes of sodium, added sugars, and saturated fatty acids (SFA) [10]–[13].

In the past, obtaining adequate iron intake in infants has been a focus area and because of this focus the prevalence of iron deficiency among infants and children has decreased in the US over the past 20–30 years. However, national data still indicate nearly one in five (18%) infants 6–12 mos falls short of the recommendations for dietary iron [14]. Therefore, iron deficiency remains a public health concern for infants, particularly because iron is a critical nutrient for neurodevelopment [15],[16]. Thus, preventing iron insufficiency remains an important priority of the American Academy of Pediatrics (AAP) [5] recommendations to prevent and treat iron deficiency for both breastfed and formula-fed infants.

The AAP recommends exclusive breast-feeding for approximately 6 mos after birth for healthy term infants. Thus, it is necessary to address the impact of breast milk in the diets of infants and toddlers for exclusively breast-fed infants given that it is the only source of nutrients. In addition, the influence of micronutrient deficiency on the development of infants who are exclusively breast fed must be addressed. While the AAP supports exclusive breastfeeding for approximately 6 mos, it recognizes that infants may be developmentally ready to initiate complementary feeding at 4 to 6 mos [5]. Introduction of solid food prior to 4 mos of age has been consistently identified as contributing to later overweight [17],[18]. If the mother cannot breast-feed, the AAP acknowledges that iron-fortified formula is an appropriate substitute for human milk. Whole cow's milk should not be introduced during the first year of life [5], because it has been linked to iron deficiency in infants and toddlers [19]. However, 20% of infants received cow's milk daily before the age of one year [20]. The recommended introduction of complementary foods (other than breast milk and infant formula) should gradually begin between 4–6 mos. However, close to 40% of infants received complementary foods at 4 mos of age [21]. After 6 mos developmentally appropriate “table foods” should be gradually introduced into the diets of toddlers. The AAP provides two practical recommendations for initiating complementary foods: (1) to introduce one “single-ingredient” food at a time, and (2) to choose foods in no particular sequence, that provide key nutrients that help meet energy needs. The AAP recommends iron-fortified infant cereal and pureed meats as good first foods because they contain protein, iron, and zinc. Iron-fortified infant rice cereal is a good choice as an infant's first complementary food for several reasons: contains important nutrients; the texture can be altered to meet an infants' developmental needs; is easily digested; and, is least likely to cause an allergic reaction [6]. By the end of the first year, a variety of foods [22] such as fruits, vegetables, and mixed dishes can be added to the diet [5].

Complementary foods consumed early in life should include a source of iron, specifically fortified infant cereal or iron-rich meat. By 4 to 6 mos, 65% of infants consumed infant cereal and small percentages consumed other types of grain products [23]. Infant cereal continued to be the predominant grain-based food in infants' diets through 9 to 11 mos. After 9 to 11 mos, the percentage of infants consuming infant cereals begins to decline and there was a marked increase in the percentage consuming other grain products and grain-based mixed dishes. Non-infant cereals were among the grain products most commonly consumed by toddlers over 12 mos [20].

Fortified rice cereal is the most commonly food introduced in the diets of infants [24]. Understanding what foods are gradually introduced in the diets after fortified rice cereal are initially introduced, will provide valuable information on complementary feeding that is based on evidence rather than based on traditions for determining best practices. In addition, understanding the nutrient profiles of food-specific patterns would be timely to inform the development of the 2020 DGA for infants and toddlers. Thus, introducing a wide variety of developmentally appropriate nutrient-rich foods takes advantage of the opportunity to instill positive initial dietary practices [24].

The objective of this study was to examine food patterns among infants and toddlers (birth to 23 mos) consuming rice baby cereal and non-rice baby cereal versus non-consumers for nutrient intake, nutrient adequacy, and food group consumption using data from NHANES 2001–2014. The results of this study have the potential to strengthen the importance of baby cereals and the introduction of complementary foods in the diet of the understudied group of infants and toddlers. The results will also add to the body of evidence considered in the development of national guidelines and healthcare professional recommendations for this population.

## 2. Methods

### 2.1. Study design, subjects, and demographics

Data from the NHANES were used to assess dietary intakes of infants and toddlers aged birth to 23 mos. The NHANES is a cross-sectional survey which uses a complex, multistage, probability sampling procedure to provide nationally representative estimates on the nutritional status of the non-institutionalized US civilian population. Full details of the sampling framework and analytical considerations can be found elsewhere [25],[26]. A proxy provided written informed consent for the infants and toddlers. The NHANES protocols were approved by the National Center for Health Statistics (NCHS) ethics review board [27]. This study did not require further institutional review as this was the analysis of secondary data without personal identifiers. To obtain an adequate sample size to produce reliable estimates within this age group, data from seven cycles (2001–2014) were combined [28],[29]. Dietary data (24-h recalls provided by parent or caregiver) from infants and toddlers less than 24 mos (n = 5,587) were used after exclusions for data judged by NCHS Staff to be unreliable (n = 544),

Most demographic information was collected via interviews using cycle appropriate questionnaires [27],[30]. Poverty Income Ratio was classified into three categories: < 1.35, 1.35 ≤ 1.85 and > 1.85. Weight, and recumbent length were obtained using the NHANES Anthropometry Procedures Manual [31]. Body mass index (BMI) was calculated as body weight (kilograms) divided by height (meters) squared. Centers for Disease Control and Prevention growth chart programs were used to determine BMI-for-age percentiles/*z*-scores; infants and toddlers with a BMI ≥ the 85^th^ but < 95^th^, and ≥ the 95^th^ percentiles were considered overweight or obese, respectively [32]. The parent/caregiver self-reported the study child's race or ethnic group according to pre-defined categories used in the NHANES.

### 2.2. Dietary intake

An in-person 24-h dietary recall was administered by trained interviewers using an Automated Multiple-Pass Method [33] with the parent/caregiver providing intakes for the infant or toddler. A second recall was collected via a telephone interview 3–10 days after the in-person interview. Food intake was assessed using USDA food codes/categories corresponding to What We Eat in America (WWEIA), the dietary component of NHANES [34]. Energy and nutrient intake from foods were determined using respective Food and Nutrient Database for Dietary Studies (FNDDS) for each NHANES cycle [35] available from total nutrient intake files. Use of supplements was not included in the analyses.

### 2.3. Statistical analyses

Three baby cereal consumption groups were examined: baby cereal non-consumers (n = 3,910) non-rice baby cereal consumers (n = 711) and rice baby cereal consumers (n = 966). Infants and toddlers who consumed both non-rice baby cereal and rice baby cereal were place in the rice baby cereal group (n = 9, 48, 61, and 10 for those 0–3, 4–6, 7–11, and 12–23 mos, respectively. Some younger subjects were exclusively breastfeed but the number of subjects exclusively breastfed dropped precipitously with age (n = 242, 47, 6, and 0 for those 0–3, 4–6, 7–11, and 12–23 mos, respectively); these subjects were placed in the baby cereal non-consumer group. Baby cereals were defined as foods in the FNDDS [35] subgroup 578 (cereals baby food) and in the WWEIA [34] category 2002 (baby food cereals). Baby foods was a separate food category. Rice cereals were baby cereal foods that contained rice in the food description. Non-rice baby cereals were baby cereal foods that did not contain rice in the food description (e.g. mixed cereal, oatmeal). Baby cereal non-consumers did not consume any type of baby cereals. There were three consumers (ages 3, 8, 23 mos) that ate “cereal, nestum” food code 56210000. Food code 56210000 is in WWEIA category 9002 but is not in FNDDS subgroup 578. This code is in FNDDS category 562 which is defined as “cooked cereals, rice.” Therefore age groups that included 3, 8, or 23 mos could show consumption of WWEIA category 9002 for subjects that were non-consumers of “baby cereals” as defined in this study. Food specific patterns were examined using both the WWEIA food subgroups and categories and the Food Patterns Equivalent Databases (FPED) main food groups [36]. The food categories used were approximately 150 WWEIA food categories along with approximately 48 subgroups and 15 main categories (excluding other and alcoholic beverages food categories). In addition, the FPED eight main food groups were assessed.

The primary focus was to determine the food-specific patterns by four age groups (i.e. 0–3 mos, 4–6mos, 7–11 mos, and 12–23 mos) for infants and toddlers consuming rice baby cereal or non-rice baby cereal versus non-consumers. Specifically, whether infants/toddlers who consumed rice baby cereal or other type of baby cereal: (1) had different food-specific patterns than non-consumers, (2) obtained more of the nutrients that are most likely to be low or deficient; and (3) consumed less added sugars, SFA, and sodium, was examined.

Food-specific patterns were defined as mean gram amounts consumed for each food group by age within the three baby cereal consumption groups. A secondary analysis focused on the impact of the food-specific patterns on nutrients (micronutrients that were most likely to be low or deficient) and nutrient adequacy (only for those ages with an Estimated Average Requirement [EAR]) [37].

Sample-weighted data were used in all statistical analyses [38], and all analyses were performed using SUDAAN Release 11.0 (Research Triangle Institute, Research Triangle Park, NC) and/or SAS 9.4 (SAS Institute, Cary, NC) to adjust the variance for the clustered sample design. The sample-weighted percentage of the population (and standard error of the percentage) who consumed baby cereals and those who did not consume baby cereals were calculated. Least-square means ± SEs were determined for nutrient intake and food group consumption by using regression analyses procedures of SAS 9.4 (Cary, NC). Covariates included age, gender, race/ethnicity, poverty income ratio, and energy intake for nutrient-related variables and food groups. P-value for statistical significance was set at p < 0.01.

Usual Intake (UI) of nutrients was determined using the preferred National Cancer Institute method [39]. The NCI macros (Mixtran and Distrib) were used to generate parameter effects after covariate adjustments and to estimate the distribution of UI. The one part NCI model was used since these substances are consumed on most days by most subjects. Covariates for these analyses were the day of the week of the 24-hr recall [coded as weekend (Friday-Sunday) or weekday (Monday-Thursday)] and sequence of dietary recall (first or second); variance estimates were obtained using the two days of intake with one-day sampling weights. Nutrient adequacy was determined as percentage below the EAR using the cut-point method [37],[40]; specifically the EAR [41] for 12–36 mos of age for iron (3.0 mg/d ); calcium (500 mg/d); zinc (2.5 mg/d); vitamins B6 (0.4 mg/d), D (10 mcg/d), E (5 mg/d), and A (210 mcg/d), were examined because they are the micronutrients that are most likely to be low or deficient in infants and toddlers [23]. The EAR for all other nutrients was used when appropriate [41].

## 3. Results

### 3.1. Sample demographics

Sample demographics of infants and toddlers 0–23 mos of age are presented in Table 1. Very few differences were shown in the demographic variables between the non-rice baby cereal consumers and the two rice baby cereal consumers. Compared to baby cereal consumers (regardless of type), baby cereal non-consumers were older, had higher mean recumbent length (cm), higher percentage of Mexican Americans, and lower percentage of formula fed infants and toddlers. Demographics for all four age groups and the three baby cereal consumption groups (i.e. baby cereal non-consumers, non-rice baby cereal consumers, and rice baby cereal consumers) are presented in Supplemental Tables 1–4. The most notable findings between baby cereal non-consumers and baby cereal consumers (regardless of type) were in the infant feeding practices. A higher percentage of baby cereal non-consumers were exclusively breast fed (ages 0–3 mos and 4–6 mos) and a lower percentage were formula fed (for all four age groups) compared to baby cereal consumers (regardless of type).

### 3.2. Nutrient intake by age groups and baby cereal consumption groups

Mean nutrient intake by age group and baby cereal consumption groups were presented in Tables 2–5. A majority of the significant differences were shown between baby cereal non-consumers and baby cereal consumers (regardless of type) across all four age groups. At 0–3 mos (Table 2), baby cereal consumers had higher mean intakes of total energy, total carbohydrate, iron, calcium, and zinc and higher mean intakes of total fat and vitamin A compared to baby cereal non-consumers. At 4–6 mos (Table 3), baby cereal consumers had significantly higher mean intakes of total energy, total carbohydrate, whole grain, vitamin B6, calcium, iron, and magnesium; as well as higher mean intakes of total fat (specifically polyunsaturated fatty acids [PUFA] and SFA) and total sugars compared to baby cereal non-consumers. At 7–11 mos (Table 4), baby cereal consumers had significantly lower mean intakes of total fat, SFA, monounsaturated fatty acids (MUFA) and total cholesterol; as well as higher mean intakes of total carbohydrate, added sugars, vitamin E, calcium, iron, zinc, and magnesium compared to baby cereal non-consumers. At 12–23 mos (Table 5), baby cereal consumers had significantly higher intakes of vitamin E, iron, and zinc compared to baby cereal non-consumers.

**Table 1.** Demographics of sample of infants and toddlers 0–23 months of age participating in the National Health and Nutrition Examination Survey (NHANES) 2001–2012.

	Total population (N= 5,587)*		**Baby Cereal Consumption Groups**					
			Baby cereal non-consumers (n = 3,910)*		Non-rice baby cereal consumers (n = 711)*		Rice baby cereal consumers (n = 966)*	
**Variables**	**Mean**	**SE**	**Mean**	**SE**	**Mean**	**SE**	**Mean**	**SE**
Age Months (mean)	11.36	0.11	12.63^a,c^	0.15	9.31^b,e^	0.27	6.68^d,f^	0.14
Males (%)	51.13	0.87	51.16	1.12	52.08	2.69	50.24	2.23
Race/Ethnicity (%)								
Mexican American	17.15	1.22	18.46^a,c^	1.31	13.15^b^	1.73	13.80^d^	1.45
Other Hispanic	7.42	0.65	7.45	0.73	8.74	1.26	6.27	0.9
Non-Hispanic White	54.73	1.85	54.43	1.94	56.1	2.86	55.14	2.68
Non-Hispanic Black	13.11	0.91	11.96^c^	0.88	14.57	1.49	17.73^d^	1.77
Other	7.58	0.54	7.71	0.64	7.43	1.13	7.06	1.01
Poverty Income Ratio (%)								
<1.35	39.98	1.15	41.03	1.34	34.39	2.57	39.21	1.98
1.35 ≤ 1.85	10.14	0.63	10.59	0.83	9.13	1.67	8.69	1.04
>1.85	49.87	1.28	48.38^a^	1.47	56.48^b^	2.66	52.09	2.23
Recumbent Length (cm )	73.78	0.17	75.14^a,c^	0.21	72.09^b,e^	0.37	68.36^d,f^	0.22
Weight for Length Percentile	57.83	0.62	57.15	0.74	59.15	1.4	60.17	1.17
Weight for Length z-score	0.26	0.02	0.23	0.02	0.3	0.05	0.33	0.04
Weight Status (%)								
Overweight	13	0.69	12.4	0.83	15.08	2.02	14.35	1.58
Overweight or Obese	21.69	0.82	20.92	1.01	24.04	2.16	23.69	1.91
Obese	8.69	0.5	8.51	0.54	8.96	1.32	9.33	1.27
Infant Feeding Practice (%)								
Breast fed, no formula	14.72	0.85	15.46	0.98	12.21	1.64	13.05	1.34
Formula, not breast fed	33.84	0.89	22.36^a,c^	0.96	59.71^b,e^	2.6	70.64^d,f^	1.92
Formula and breast fed	5.55	0.38	4.46^c^	0.36	6.13	1.05	10.52^d^	1.37
WIC Participant (%)	48.8	1.35	48.48	1.49	46.62	2.89	52.14	2.26
WIC Eligible (%)	58.85	1.31	59.61	1.48	54.11	2.75	58.79	2.24

Abbreviations: SE: Standard error, WIC: Women, Infants and Children. *Sample sizes for the various variables were slightly smaller due to missing data. ^a,b^ Means with different superscripts indicate significant differences (p < 0.01) between baby cereal non-consumers and non-rice cereal consumers. ^c,d^ Means with different superscripts indicate significant differences (p < 0.01) between baby cereal non-consumers and baby rice cereal consumers. ^e,f^ Means with different superscripts indicate significant differences (p < 0.01) between non-rice cereal consumers and baby rice cereal consumers.

**Table 2.** Nutrient intake for infants 0–3 months participating in National Health and Nutrition Examination Survey (NHANES) 2001–2012 by baby cereal consumption groups.

	Total Population (N=1,065)*		**Baby Cereal Consumption Groups**					
			Baby cereal non-consumers (n=859)*		Non-rice baby cereal consumers (n=51)*		Rice baby cereal consumers (n=155)*	
**Macronutrients**	**LSM**	**SE**	**LSM**	**SE**	**LSM**	**SE**	**LSM**	**SE**
Total energy (Kcal)	387	13.9	348^a,c^	16.2	529^b^	32.0	567^d^	31.5
Total fat (gm)	20.1	0.1	20.5^a,c^	0.1	18.5^b^	0.5	17.7^d^	0.2
SFA (gm)	8.2	0.1	8.4^c^	0.1	7.4	0.6	7.0^d^	0.2
MUFA (gm)	6.9	0.1	7.0^c^	0.1	6.4	0.5	6.3^d^	0.2
PUFA (gm)	4.2	0.0	4.3^c^	0.0	4.1	0.1	3.8^d^	0.1
Cholesterol (mg)	8.3	0.3	8.8	0.4	5.4	1.4	6.1	1.1
Total carbohydrate (gm)	43.3	0.1	42.3^a,c^	0.1	47.2^b^	1.2	48.1^d^	0.5
Refined grains (oz eq)	0.1	0.0	0.0^c^	0.0	0.0^e^	0.0	0.5^d,f^	0.0
Whole grains (oz eq)	0.0	0.0	0.0^a^	0.0	0.3^b,e^	0.1	0.0^f^	0.0
Dietary fiber (gm)	0.1	0.0	0.1^a^	0.0	1.1^b,e^	0.2	0.3^f^	0.1
Total sugars (gm)	39.4	0.2	40.5^c^	0.2	37.9^e^	1.1	33.8^d,f^	0.7
Added sugars (tsp eq)	0.0	0.0	0.0	0.0	0.1	0.1	0.0	0.0
**Micronutrients**								
Vitamin B6 (mg)	0.3	0.0	0.2^c^	0.0	0.3	0.0	0.3^d^	0.0
Vitamin D (D2+D3) (mcg)	5.6	0.1	5.6	0.1	4.7	0.6	5.5	0.2
Vitamin E as alpha-tocopherol (mg)	4.8	0.1	4.8	0.1	4.6	0.3	4.9	0.2
Vitamin A, RAE (mcg)	339.6	3.6	349.3^a,c^	4.3	277.9^b^	16.9	301.8^d^	7.8
Calcium (mg)	345.5	2.8	332.8^c^	2.6	366.3	17.9	409.6^d^	10.3
Iron (mg)	8.0	0.1	6.9^a,c^	0.1	11.3^b^	1.0	13.3^d^	0.5
Zinc (mg)	3.4	0.0	3.4	0.0	3.5	0.2	3.4	0.1
Magnesium (mg)	38.1	0.5	34.1^a,c^	0.4	41.7^b,e^	2.9	59.1^d,f^	2.2
Potassium (mg)	427.8	2.4	426.5	2.9	438.4	9.3	432.7	7.8

Abbreviations: LSM: Least square mean, SE: Standard error, SFA: Saturated fatty acids, MUFA: Monounsaturated fatty acids, PUFA: Polyunsaturated fatty acids, oz eq: ounce equivalent, tsp eq: teaspoon equivalent. *Sample sizes for the various variables were slightly smaller due to missing data. ^a,b^ Means with different superscripts indicate significant differences (p < 0.01) between baby cereal non-consumers and non-rice cereal consumers. ^c,d^ Means with different superscripts indicate significant differences (p < 0.01) between baby cereal non-consumers and baby rice cereal consumers. ^e,f^ Means with different superscripts indicate significant differences (p < 0.01) between non-rice cereal consumers and baby rice cereal consumers.

**Table 3.** Nutrient intake for 4–6 months of age by baby cereal consumption groups.

	Total Population (N = 807)*		**Baby Cereal Consumption Groups**					
			Baby cereal non-consumers (n = 323)*		Non-rice baby cereal consumers (n = 167)*		Rice baby cereal consumers (n = 317)*	
**Macronutrients**	**LSM**	**SE**	**LSM**	**SE**	**LSM**	**SE**	**LSM**	**SE**
Total energy (Kcal)	569	16.1	455^a,c^	25.7	588^b^	36.0	666^d^	23.3
Total fat (gm)	24.3	0.2	26.3^a,c^	0.3	22.9^b^	0.6	23.2^d^	0.4
SFA (gm)	9.9	0.1	11.1^a,c^	0.2	8.9^b^	0.3	9.3^d^	0.2
MUFA (gm)	8.4	0.1	8.9^c^	0.2	8.2	0.3	8.0^d^	0.2
PUFA (gm)	5.1	0.1	5.5^a,c^	0.1	4.9^b^	0.1	5.0^d^	0.1
Cholesterol (mg)	13.9	1.0	17.8^c^	1.7	14.1	3.1	10.0^d^	1.1
Total carbohydrate (gm)	73.0	0.6	68.5^a,c^	0.7	76.1^b^	1.4	75.6^d^	0.9
Refined grains (oz eq)	0.4	0.0	0.1^c^	0.0	0.2^e^	0.0	0.7^d,f^	0.0
Whole grains (oz eq)	0.2	0.0	0.0^a,c^	0.0	0.6^b,e^	0.1	0.1^d,f^	0.0
Dietary fiber (gm)	2.3	0.1	1.8^a^	0.2	3.8^b,e^	0.3	2.1^f^	0.1
Total sugars (gm)	54.2	0.5	57.0^a,c^	0.9	52.7 ^b^	1.0	52.3^d^	0.7
Added sugars (tsp eq)	0.3	0.1	0.3	0.1	0.4	0.2	0.2	0.1
**Micronutrients**								
Vitamin B6 (mg)	0.4	0.0	0.4^a,c^	0.0	0.5^b^	0.0	0.5^d^	0.0
Vitamin D (D2+D3) (mcg)	6.8	0.1	7.0	0.1	6.7	0.3	6.8	0.2
Vitamin E as alpha-tocopherol (mg)	6.6	0.1	6.6	0.2	6.9	0.4	6.4	0.1
Vitamin A, RAE (mcg)	508.9	10.3	526.2	14.9	500.7	24.0	497.1	14.6
Calcium (mg)	496.8	7.7	436.5^a,c^	7.5	527.7^b^	20.7	536.4^d^	10.7
Iron (mg)	14.1	0.3	9.4^a,c^	0.3	17.2^b^	0.6	16.9^d^	0.5
Zinc (mg)	4.8	0.1	4.5^a^	0.1	5.3^b,e^	0.2	4.7^f^	0.1
Magnesium (mg)	70.5	1.1	57.5^a,c^	1.0	76.4^b^	2.0	79.5^d^	1.9
Potassium (mg)	723.2	6.4	705.8^a^	11.0	756.0^b^	14.7	721.0	12.0

Abbreviations: LSM: Least square mean, SE: Standard error, SFA: Saturated fatty acids, MUFA: Monounsaturated fatty acids, PUFA: Polyunsaturated fatty acids, oz eq: ounce equivalent, tsp eq: teaspoon equivalent. *Sample sizes for the various variables were slightly smaller due to missing data. ^a,b^ Means with different superscripts indicate significant differences (p < 0.01) between baby cereal non-consumers and non-rice cereal consumers. ^c,d^ Means with different superscripts indicate significant differences (p < 0.01) between baby cereal non-consumers and baby rice cereal consumers. ^e,f^ Means with different superscripts indicate significant differences (p < 0.01) between non-rice cereal consumers and baby rice cereal consumers.

**Table 4.** Nutrient intake for 7–11 months of age by baby cereal consumption groups.

	Total Population (N = 1,342)*		**Baby Cereal Consumption Groups**					
			Baby cereal non-consumers (n = 633)*		Non-rice baby cereal consumers (n = 338)*		Rice baby cereal consumers (n = 371)*	
**Macronutrients**	**LSM**	**SE**	**LSM**	**SE**	**LSM**	**SE**	**LSM**	**SE**
Total energy (Kcal)	843	11.9	831	20.5	857	21.9	847	19.6
Total fat (gm)	32.7	0.3	34.3^a,c^	0.4	31.5^b^	0.3	31.5^d^	0.5
SFA (gm)	13.0	0.2	14.0^a,c^	0.3	12.3^b^	0.3	12.3^d^	0.2
MUFA (gm)	11.0	0.1	11.6^a,c^	0.2	10.6^b^	0.2	10.7^d^	0.2
PUFA (gm)	6.5	0.1	6.5	0.1	6.5	0.1	6.6	0.1
Cholesterol (mg)	62.6	2.6	84.8^a,c^	5.2	46.4^b^	4.6	46.0^d^	2.7
Total carbohydrate (gm)	114.7	0.7	108.8^a,c^	1.1	119.0^b^	0.9	119.0^d^	1.2
Refined grains (oz eq)	1.2	0.0	1.2^a^	0.1	0.9^b,e^	0.1	1.4^f^	0.1
Whole grains (oz eq)	0.4	0.0	0.2^a^	0.0	0.8^b,e^	0.0	0.3^f^	0.0
Dietary fiber (gm)	5.8	0.1	5.3^a^	0.2	6.9^b,e^	0.2	5.3^f^	0.2
Total sugars (gm)	72.7	0.6	71.3	1.0	75.0	1.1	72.5	1.0
Added sugars (tsp eq)	1.7	0.1	2.3^a,c^	0.1	1.1^b^	0.1	1.2^d^	0.1
**Micronutrients**								
Vitamin B6 (mg)	0.8	0.0	0.8	0.0	0.8	0.0	0.8	0.0
Vitamin D (D2+D3) (mcg)	7.5	0.1	7.1^c^	0.2	7.8	0.2	7.9^d^	0.2
Vitamin E as alpha-tocopherol (mg)	6.8	0.1	5.9^a,c^	0.2	7.5^b^	0.2	7.4^d^	0.2
Vitamin A, RAE (mcg)	615.0	9.9	588.7	14.5	643.5	20.1	625.2	20.7
Calcium (mg)	673.9	8.5	630.0^a,c^	11.8	694.3^b^	13.3	717.8^d^	14.9
Iron (mg)	15.6	0.3	9.6^a,c^	0.2	20.1^b^	0.6	20.1^d^	0.6
Zinc (mg)	6.2	0.1	5.6^a,c^	0.1	7.0^b,e^	0.2	6.2^d,f^	0.1
Magnesium (mg)	114.0	1.0	104.2^a,c^	1.4	117.2^b^	1.9	125.1^d^	2.5
Potassium (mg)	1,209.4	9.3	1,208.0	17.3	1,233.1	19.6	1,188.1	13.6

Abbreviations: LSM: Least square mean, SE: Standard error, SFA: Saturated fatty acids, MUFA: Monounsaturated fatty acids, PUFA: Polyunsaturated fatty acids, oz eq: ounce equivalent, tsp eq: teaspoon equivalent. *Sample sizes for the various variables were slightly smaller due to missing data. ^a,b^ Means with different superscripts indicate significant differences (p <0.01) between baby cereal non-consumers and non-rice cereal consumers. ^c,d^ Means with different superscripts indicate significant differences (p <0.01) between baby cereal non-consumers and baby rice cereal consumers. ^e,f^ Means with different superscripts indicate significant differences (p <0.01) between non-rice cereal consumers and baby rice cereal consumers.

**Table 5.** Nutrient intake for 12–23 months of age by baby cereal consumption groups.

	Total Population (N = 2,010)*		**Baby Cereal Consumption Groups**					
			Baby cereal non-consumers (n = 1,846)*		Non-rice baby cereal consumers (n = 104)*		Rice baby cereal consumers (n = 60)*	
**Macronutrients**	**LSM**	**SE**	**LSM**	**SE**	**LSM**	**SE**	**LSM**	**SE**
Total energy (Kcal)	1,257	16.3	1,261	17.2	1,209	47.9	1,187	81.9
Total fat (gm)	47.0	0.3	47.4^a^	0.3	41.1^b,e^	0.9	45.9^f^	1.5
SFA (gm)	19.2	0.2	19.3^a^	0.2	16.7^b,e^	0.5	20.3^f^	0.8
MUFA (gm)	15.5	0.1	15.7^a^	0.1	13.3^b^	0.4	14.3	0.6
PUFA (gm)	7.9	0.1	8.0	0.1	7.1	0.3	7.3	0.5
Cholesterol (mg)	167.3	3.8	170.2^a^	4.1	124.5^b^	7.9	138.8	16.8
Total carbohydrate (gm)	165.1	0.9	164.1^a^	0.9	182.6^b^	2.6	171.0	3.7
Refined grains (oz eq)	2.7	0.1	2.8	0.1	2.5	0.2	2.3	0.2
Whole grains (oz eq)	0.5	0.0	0.5^a^	0.0	1.1^b,e^	0.1	0.5^f^	0.1
Dietary fiber (gm)	8.6	0.1	8.5^a,c^	0.1	10.8^b,e^	0.51	7.1^d,f^	0.4
Total sugars (gm)	94.7	0.8	94.1^c^	0.8	99.8	3.5	107.2^d^	3.1
Added sugars (tsp eq)	7.0	0.2	7.1	0.2	6.44	0.8	6.3	0.7
**Micronutrients**								
Vitamin B6 (mg)	1.2	0.0	1.2	0.0	1.2	0.1	1.2	0.1
Vitamin D (D2+D3) (mcg)	8.2	0.1	8.2^c^	0.1	8.1^e^	0.4	11.1^d,f^	0.5
Vitamin E as alpha-tocopherol (mg)	3.8	0.1	3.7^a,c^	0.1	5.0^b^	0.3	5.7^d^	0.6
Vitamin A, RAE (mcg)	545.9	9.9	544.1	10.3	546.5	42.4	629.1	43.8
Calcium (mg)	1012.1	10.8	1007.6^c^	11.4	1005.7^e^	31.9	1236.8^d,f^	50.8
Iron (mg)	9.4	0.1	8.7^a,c^	0.1	18.9^b^	1.2	17.3^d^	1.5
Zinc (mg)	7.2	0.1	7.1^a,c^	0.1	8.1^b^	0.4	8.3^d^	0.5
Magnesium (mg)	174.4	1.1	173.1^a^	1.2	190.7^b^	6.1	191.2	7.8
Potassium (mg)	1916.4	12.5	1914.0	12.8	1934.6	58.9	1981.1	56.2

Abbreviations: LSM: Least square mean, SE: Standard error, SFA: Saturated fatty acids, MUFA: Monounsaturated fatty acids, PUFA: Polyunsaturated fatty acids, oz eq: ounce equivalent, tsp eq: teaspoon equivalent. *Sample sizes for the various variables were slightly smaller due to missing data. ^a,b^ Means with different superscripts indicate significant differences (p < 0.01) between baby cereal non-consumers and non-rice cereal consumers. ^c,d^ Means with different superscripts indicate significant differences (p < 0.01) between baby cereal non-consumers and baby rice cereal consumers. ^e,f^ Means with different superscripts indicate significant differences (p < 0.01) between non-rice cereal consumers and baby rice cereal consumers.

### 3.3. Nutrient adequacy by age groups and baby cereal consumption groups

The percentage of the population below the EAR for those 12–23 mos (only age group with EARs) was very low (< 2% for 13 of the nutrients evaluated (iron; zinc; copper; folate; magnesium; niacin; phosphorous; riboflavin; selenium; thiamin; and vitamins A, E, D, B_6_, B_12_, and C) (data not shown). Approximately 5.4 ± 0.8%, 3.3 ± 0.34%, 71.2 ± 1.4%, and 79.7 ± 1.8% of those 12–23 mos had intakes of calcium, iron, and vitamins D and E below the EAR, respectively. While there were no significant differences in nutrient adequacy when comparing rice baby cereal consumers with non-rice baby cereals, consumers of rice baby cereal had a significantly lower percentage of the population below the EAR for calcium (5.8 ± 0.8 v 0.0 ± 0.8) and vitamin E (81.2 ± 1.7 v 54.2 ± 7.4). Additionally, as compared to baby cereal non-consumers, both baby cereal consumption groups had significantly lower percentages of the population below the EAR for iron (3.6 ± 0.4, 0.04 ± 0.1, and 0.6 ± 0.5 % for baby cereal non-consumers, non-rice baby cereal consumers, and rice baby cereal consumer, respectively).

### 3.4. Food group consumption (FPED main food groups) by age groups and baby cereal consumption groups

Mean food group (eight FPED main food groups) consumption by age groups and by baby cereal consumption groups are presented in Tables 6–9. The majority of the significant differences were shown between baby cereal non-consumers and baby cereal consumers (regardless of type) across all four age groups. At 0–3 mos (Table 6), baby cereal consumers had higher mean intake of total grains than baby cereal non-consumers. At 4–6 mos (Table 7), baby cereal consumers had higher mean intake of total grains specifically whole grains, compared to baby cereal non-consumers. At 7–11 mos (Table 8), baby cereal consumers had lower mean intakes of protein foods, total dairy, solid fats and added sugars and oils compared to baby cereal non-consumers. At 12–23 mos (Table 9), the majority of the significant differences were shown between baby cereal non-consumers and the two types of baby cereal consumers (i.e. non-rice baby cereal consumers and rice baby cereal consumers). Baby cereal consumers had higher mean intakes of total dairy, solid fats, and oils compared to non-rice baby cereal non-consumers. Baby cereal consumers had lower mean intakes of protein foods and oils than rice baby cereal non-consumers. Rice baby cereal consumers had significantly lower mean intakes of vegetables and total grains (specifically whole grains) compared to non-rice baby cereal consumers.

### 3.5. Food group consumption (WWEIA food subgroups and food categories) by age groups and baby cereal consumption groups

Mean food group (WWEIA food subgroups and food categories) consumption by age groups and by baby cereal consumption groups are presented in Supplemental Tables 5–12.

**Table 6.** Food group consumption for 0–3 months of age by baby cereal consumption groups.

	Total population (N = 1,065)*		**Baby Cereal Consumption Groups**					
			Baby cereal non-consumers (n = 859)*		Non-rice baby cereal consumers (n = 51)*		Rice baby cereal consumers (n = 155)*	
**Food Groups**	**LSM**	**SE**	**LSM**	**SE**	**LSM**	**SE**	**LSM**	**SE**
Protein Foods (oz eq)	0.0	0.0	0.0	0.0	0.0	0.0	0.0	0.0
Total dairy (cup eq)	0.0	0.0	0.0	0.0	0.0	0.0	0.0	0.0
Total Vegetables (cup eq)**	0.0	0.0	0.0	0.0	0.0	0.0	0.0	0.0
Legumes (oz eq)***	0.0	.***	0.0	.***	0.0	.***	0.0	.***
Total Fruits (cup eq)	0.0	0.0	0.0	0.0	0.1	0.0	0.0	0.0
Grains (oz eq)	0.1	0.0	0.0^a,c^	0.0	0.4^b^	0.1	0.6^d^	0.0
Whole grains	0.0	0.0	0.0^a^	0.0	0.3^b,e^	0.1	0.0^f^	0.0
Refined grains	0.1	0.0	0.0^c^	0.0	0.0^e^	0.0	0.5^d,f^	0.0
Sofas (gm)	0.1	0.0	0.1	0.0	0.5	0.3	0.1	0.1
Solid fats (gm)	0.0	0.0	0.0	0.0	0.0	0.0	0.1	0.1
Added Sugars (tsp eq)	0.0	0.0	0.0	0.0	0.1	0.1	0.0	0.0
Oils (gm)	0.0	0.0	0.0	0.0	0.0	0.0	0.0	0.0

Abbreviations: LSM: Least square mean, SE: Standard error, oz eq: Ounce equivalent, cup eq: Cup equivalent, tsp eq: teaspoon equivalent. *Sample sizes for the various variables were slightly smaller due to missing data. ** Excluding legumes. ***All subjects have zero consumption and thus there are no variance estimates. ^a,b^ Means with different superscripts indicate significant differences (p < 0.01) between baby cereal non-consumers and non-rice cereal consumers. ^c,d^ Means with different superscripts indicate significant differences (p < 0.01) between baby cereal non-consumers and baby rice cereal consumers. ^e,f^ Means with different superscripts indicate significant differences (p < 0.01) between non-rice cereal consumers and baby rice cereal consumers.

**Table 7.** Food group consumption for 4-6 months of age by baby cereal consumption groups.

	Total population (N = 807)*		**Baby Cereal Consumption Groups**					
			Baby cereal non-consumers (n = 323)*		Non-rice baby cereal consumers (n = 167)*		Rice baby cereal consumers (n = 317)*	
**Food Groups**	**LSM**	**SE**	**LSM**	**SE**	**LSM**	**SE**	**LSM**	**SE**
Protein Foods (oz eq)	0.1	0.0	0.1	0.0	0.1	0.0	0.1	0.0
Total dairy (cup eq)	0.0	0.0	0.0	0.0	0.0	0.0	0.0	0.0
Total Vegetables (cup eq)**	0.2	0.0	0.2	0.0	0.2	0.0	0.2	0.0
Legumes (oz eq)	0.0	0.0	0.0	0.0	0.0	0.0	0.0	0.0
Total Fruits (cup eq)	0.3	0.0	0.3	0.0	0.4	0.0	0.3	0.0
Grains (oz eq)	0.6	0.0	0.2^a,c^	0.0	0.8^b^	0.1	0.8^d^	0.0
Whole grains	0.2	0.0	0.0^a,c^	0.0	0.6^b,e^	0.1	0.1^d,f^	0.0
Refined grains	0.4	0.0	0.1^c^	0.0	0.2^e^	0.0	0.7^d,f^	0.0
Sofas (gm)	1.6	0.3	1.7	0.3	2.2	0.8	1.1	0.3
Solid fats (gm)	0.4	0.1	0.6	0.1	0.5	0.2	0.3	0.1
Added Sugars (tsp eq)	0.3	0.1	0.3	0.1	0.4	0.2	0.2	0.1
Oils (gm)	0.3	0.1	0.3	0.1	0.6	0.2	0.2	0.0

Abbreviations: LSM: Least square mean, SE: Standard error, oz eq: Ounce equivalent, cup eq: Cup equivalent, tsp eq: teaspoon equivalent. *Sample sizes for the various variables were slightly smaller due to missing data. ** Excluding legumes. ^a,b^ Means with different superscripts indicate significant differences (p < 0.01) between baby cereal non-consumers and non-rice cereal consumers. ^c,d^ Means with different superscripts indicate significant differences (p < 0.01) between baby cereal non-consumers and baby rice cereal consumers. ^e,f^ Means with different superscripts indicate significant differences (p < 0.01) between non-rice cereal consumers and baby rice cereal consumers.

**Table 8.** Food group consumption for 7–11 months of age by baby cereal consumption groups.

	Total population (N = 1,342)*		**Baby Cereal Consumption Groups**					
			Baby cereal non-consumers (n = 633)*		Non-rice baby cereal consumers (n = 338)*		Rice baby cereal consumers (n = 371)*	
Food Groups*	**LSM**	**SE**	**LSM**	**SE**	**LSM**	**SE**	**LSM**	**SE**
Protein Foods (oz eq)	0.7	0.0	0.9^a,c^	0.1	0.6^b^	0.0	0.6^d^	0.1
Total dairy (cup eq)	0.6	0.0	0.9^a,c^	0.1	0.5^b^	0.1	0.4^d^	0.0
Total Vegetables (cup eq)**	0.4	0.0	0.4	0.0	0.5	0.0	0.5	0.0
Legumes (oz eq)	0.1	0.0	0.1	0.0	0.1	0.0	0.1	0.0
Total Fruits (cup eq)	0.8	0.0	0.7^a^	0.0	0.9^b^	0.0	0.8	0.0
Grains (oz eq)	1.6	0.0	1.5	0.1	1.6	0.1	1.7	0.1
Whole grains	0.4	0.0	0.2^a^	0.0	0.8^b,e^	0.0	0.3^f^	0.0
Refined grains	1.2	0.0	1.2^a^	0.1	0.9^b,e^	0.1	1.4^f^	0.1
Sofas (gm)	13.7	0.4	19.1^a,c^	0.8	9.4^b^	0.8	9.8^d^	0.6
Solid fats (gm)	7.0	0.3	9.8^a,c^	0.4	5.0^b^	0.4	5.0^d^	0.3
Added Sugars (tsp eq)	1.7	0.1	2.3^a,c^	0.1	1.1^b^	0.1	1.2^d^	0.1
Oils (gm)	2.6	0.1	3.5^a,c^	0.3	1.8^b^	0.2	1.9^d^	0.1

Abbreviations: LSM: Least square mean, SE: Standard error, oz eq: Ounce equivalent, cup eq: Cup equivalent, tsp eq: teaspoon equivalent. *Sample sizes for the various variables were slightly smaller due to missing data. ** Excluding legumes. ^a,b^ Means with different superscripts indicate significant differences (p < 0.01) between baby cereal non-consumers and non-rice cereal consumers. ^c,d^ Means with different superscripts indicate significant differences (p < 0.01) between baby cereal non-consumers and baby rice cereal consumers. ^e,f^ Means with different superscripts indicate significant differences (p < 0.01) between non-rice cereal consumers and baby rice cereal consumers.

**Table 9.** Food group consumption for 12–23 months of age by baby cereal consumption groups.

	Total population (N = 2,010)*		**Baby Cereal Consumption Groups**					
			Baby cereal non-consumers (n = 1,846)*		Non-rice baby cereal consumers (n = 104)*		Rice baby cereal consumers (n = 60)*	
**Food Groups**	**LSM**	**SE**	**LSM**	**SE**	**LSM**	**SE**	**LSM**	**SE**
Protein Foods (oz eq)	2.0	0.0	2.1^c^	0.1	1.7	0.2	1.3^d^	0.1
Total dairy (cup eq)	2.7	0.0	2.7^a^	0.0	2.1^b^	0.2	2.8	0.3
Total Vegetables (cup eq)**	0.5	0.0	0.5^c^	0.0	0.7^e^	0.1	0.4^d,f^	0.1
Legumes (oz eq)	0.2	0.0	0.2	0.0	0.1	0.0	0.1	0.1
Total Fruits (cup eq)	1.4	0.0	1.3	0.0	1.6	0.1	1.4	0.1
Grains (oz eq)	3.2	0.1	3.2	0.1	3.6^e^	0.2	2.8^f^	0.2
Whole grains	0.5	0.0	0.5^a^	0.0	1.1^b,e^	0.1	0.5^f^	0.1
Refined grains	2.7	0.1	2.8	0.1	2.5	0.2	2.3	0.2
Sofas (gm)	54.4	0.8	55.0^a^	0.9	45.7^b^	3.4	48.9	3.4
Solid fats (gm)	26.3	0.3	26.6^a^	0.3	20.0^b^	1.1	23.9	1.9
Added Sugars (tsp eq)	7.0	0.2	7.1	0.2	6.4	0.8	6.3	0.7
Oils (gm)	8.9	0.2	9.2^a,c^	0.2	6.4^b^	0.7	4.9^d^	0.8

Abbreviations: LSM: Least square mean, SE: Standard error, oz eq: Ounce equivalent, cup eq: Cup equivalent, tsp eq: teaspoon equivalent. *Sample sizes for the various variables were slightly smaller due to missing data. ** Excluding legumes. ^a,b^ Means with different superscripts indicate significant differences (p < 0.01) between baby cereal non-consumers and non-rice cereal consumers. ^c,d^ Means with different superscripts indicate significant differences (p < 0.01) between baby cereal non-consumers and baby rice cereal consumers. ^e,f^ Means with different superscripts indicate significant differences (p < 0.01) between non-rice cereal consumers and baby rice cereal consumers.

#### 3.5.1. Food subgroups

At 0–3 mos (Supplemental Table 5), baby cereal non-consumers had lower mean intakes of infant formulas and baby foods compared to the two baby cereal consumption groups. Baby cereal consumers had lower mean intake of water compared to rice baby cereal consumers. At 4–6 mos (Supplemental Table 6), baby cereal non-consumers had lower mean intakes of baby foods and water compared to the two baby cereal consumption groups. Baby cereal non-consumers had lower mean intake of infant formulas compared to rice baby cereal consumers. At 7–11 mos (Supplemental Table 7), the majority of the significant differences were found between baby cereal non-consumers and the two baby cereal consumption groups. Baby cereal non-consumers had lower mean intakes of infant formulas and baby foods than the two baby cereal consumption groups. In addition, baby cereal non-consumers had higher mean intakes of milk, yogurt, grain-based mixed dishes, fruits, eggs, poultry, cured meats/poultry, vegetables (excluding white potatoes), cooked cereals, breads, rolls, tortillas, ready-to-eat cereals, sweet bakery products, and condiments and sauces than the two baby cereal consumption groups. At 12–23 mos (Supplemental Table 8), baby cereal non-consumers had lower mean intakes of infant formulas than the two baby cereal consumption groups. Baby cereal non-consumers had higher mean intakes of cheese, pizza, sandwiches, cured meats/poultry, desserts, and fats and oils than the two baby cereal consumption groups.

#### 3.5.2. Food categories

Excluding cereal classifications, significant differences in the consumption of foods by food categories only applied to the ages 7–11 mos (Supplemental Table 9) and 12–23 mos (Supplemental Table 10). At 7–11 mos, baby cereal non-consumers had lower mean intakes of baby food-fruit, baby food-meat and dinners, and baby food-vegetables than the two baby cereal consumption groups. Baby cereal non-consumers had higher mean intakes of whole milk and reduced fat milk, pasta mixed dishes (excluding macaroni and cheese), eggs and omelets, chicken-whole pieces, nuggets, patties and tenders, oatmeal, yeast breads, higher sugar (> 21 gm/100 gm) ready-to-eat cereal, and cookies and brownies. At 12–23 mos, baby cereal non-consumers had higher mean intakes of hamburgers, frankfurters, peanut butter and jelly sandwiches, egg breakfast sandwiches, sausages, bacon, and ice cream and frozen dairy desserts compared to the two baby cereal consumption groups.

## 4. Discussion

Very few differences were found in intakes of nutrients and food group consumption between rice baby cereal consumers and non-rice baby cereal consumers. However there were numerous significant differences when each baby cereal group was compared to non-consumers. While there were some differences specific to certain age groups, typically intakes of carbohydrates, iron, calcium, magnesium, zinc, and vitamin E were higher in baby-cereal consumers. The higher intakes in the baby cereal consumer groups as compared to baby cereal non-consumers also led to a significantly lower percentage of the population with intakes below the EAR for iron, calcium, and vitamin E.

The AAP has recommended exclusive breast feeding during the first 6 mos of life, and continued breast feeding with appropriate complementary food until 2 years [5],[42]. Breast milk is a nutritionally complete diet that provides adequate nutrients for infants during the first 6 mos, and if mothers cannot breast feed, iron-fortified formula is an appropriate substitute for human milk. Whole cow's milk should not be introduced during the first year of age. Introduction of complementary foods should be gradually introduced between 4–6 mos of age especially during the high risk period of 4–6 mos of life [5],[42]. Introduction of solid food prior to 4 mos has been consistently identified as contributing to later overweight. After 6 mos, developmentally appropriate complementary food should be gradually introduced in the diets of toddlers. The AAP recommends iron-fortified cereals and pureed meats as good first foods because they contain protein, iron, and zinc. By the end of the first year, a variety of foods, in no particular sequence, such as fruits, vegetables, and mixed dishes can be added to the complementary diet [5],[42] of the child. Some suggest introducing vegetables before fruits so the toddler will develop a taste for vegetables. Toddlers already have an innate sweet preference; thus, fruit is likely to be more readily accepted than vegetables, which are sometimes bitter. The increased preference for sweet taste during early development is universal [43]–[50].

Key findings in this investigation, which will need to be confirmed in other studies indicated the majority of the significant differences were shown between the baby cereal non-consumers and the two baby cereal consumption groups. Baby cereal non-consumers had a significantly higher percentage exclusively breast feeding at ages 0–3 mos and a lower percentage formula feeding at all four age groups. Infants 0–3 mos and 4–6 mos in both baby cereal consumption groups consumed solid foods. These included baby foods, baby beverages, sweetened beverages, coffee and tea, 100% juice, vegetables (excluding white potatoes), fruit, sugars, milk and yogurt, and mixed dishes-soups.

Baby cereal non-consumers had significantly lower intakes of baby foods-meat and dinners and baby-food vegetables at 7–11 mos and 12–23 mos of age. The two baby cereal consumption groups and the non-consumers had intakes aligned with the “American diet” (i.e. increased variety of foods; table and fast foods).

For infants and toddlers, the micronutrients that are most likely to be low or deficient (depending on the developmental ages and the introduction of complementary and table foods) are dietary fiber; iron; calcium; magnesium; potassium; vitamins B6, D, E and A; as well as intakes high in SFA, added sugars, and sodium [10],[12],[13]. In this study, intake of iron (3.3%), calcium (5.4%), and vitamins D (71.2%) and E (79.7%) were below the EAR. For zinc, magnesium, phosphorous, and vitamins A and C < 1% of 12–23 mos olds were below the EAR. Demmer et al [11] reported inadequate intakes of calcium (2.3%) and vitamins D (78%) and vitamin E (66%). In both of these studies dietary supplement intake was not included in the analyses, which may have impacted nutrient adequacy. It has been reported that 18.2% of infants and toddlers used ≥ one dietary supplement in the past 30 days. Use was lower among infants than among toddlers [51]. Significant differences were shown among the baby cereal non-consumers and the two baby cereal consumption groups compared to baby cereal consumers. At 0–3 mos, baby cereal non-consumers had significantly higher intakes of vitamin A and lower intakes of iron, calcium, and magnesium. At 4–6 mos, baby cereal non-consumers had lower intakes of iron, calcium, magnesium and vitamin B6 and higher intakes of SFA compared to the two baby cereal consumption groups. At 7–11 mos, baby cereal non-consumers had lower intakes of SFA, added sugars, iron, zinc, magnesium and vitamin E, compared to the two baby cereal consumption groups. At 12–23 mos, baby cereal non-consumers had lower mean intakes of iron and zinc and vitamin E, compared to the two baby cereal consumption groups. The differences in iron and vitamin E were such as to impact the percentage of the population below the EAR the percentage of the population below the EAR. For iron and vitamin E dropped from about 3.5% to < 1% and about 80 % to about 60%, respectively. This suggests that about 140,000 infants/toddlers would no longer have inadequate intakes of iron and over 773,000 infants/toddlers would no longer have inadequate intakes of vitamin E if baby cereals were consumed. Fortification of baby cereals is likely responsible for these intake differences. The lower intakes of iron (at all four age groups) and zinc (at ages 7–11 mos and 12–23 mos) is problematic among non-consumers of baby cereal and may reflect that a higher percentage is breast feeding and had lower intakes of baby foods-meat and dinners being introduced in the diet. Given that breast milk was not quantified in the diets of the infants, the lower intake of total energy, zinc, and iron may be an under-estimation of several nutrients and is a major limitation of this study. It is important to continue monitoring intakes of nutrients and the types of complementary foods being introduced in the diet and when they are introduced. Iron and zinc deficiencies are not uncommon in older breastfed infants [5]. Thus, routine screening for anemia and zinc deficiency during the second year of life may be needed [52],[53].

The major strengths of this study include the use of a nationally representative sample of U.S. infants and toddlers and the robust and standardized collection of dietary data within the NHANES and the use of usual intake techniques to assess nutrient adequacy. To determine food group contributions the WWEI food group categories were used. An advantage of this food group classification system is that similar foods and beverages are grouped together based on usage and nutrient content, which can be useful for providing practical advice to parents and health professionals about the types of foods that should be encouraged in the diets of infants and toddlers. Alternatively, as this food group classification system does not disaggregate food items into ingredients, comparisons with previous work is limited [54]. Other limitations include the potential for parents/caregivers to over or under-report foods consumed by their child in addition to the exclusion of supplement intake in the analysis, which may have impacted nutrient adequacy. Comparison with other published study results [11],[14],[20],[21],[24],[42],[54] is very limited due to lack of consistency in study design, survey years, age groups used, and statistical approaches.

## 5. Conclusion

In conclusion, this study provides detailed information on the introduction of baby cereals and other complementary foods and intakes of nutrients that require special attention during early life. This information can be used to inform targeted dietary strategies within public health initiatives to improve the diets of infants and toddlers and to guide parents regarding appropriate food selection. Finally, this information is useful to monitor changes in eating habits of U.S. infants and toddlers over time.
