# Supplementary material for: Nutrient intake, introduction of baby cereals and other complementary foods in the diets of infants and toddlers from birth to 23 months of age
Source: AIMS Public Health. 2020 Mar 4;7(1):123–47. doi: 10.3934/publichealth.2020012 (PMC7109529; doi:10.3934/publichealth.2020012)
Supplement: Supplementary file 1 [file publichealth-07-01-012-s001.pdf]

AIMS Public Health

AIMS Public Health, 7(1): 123–147.
DOI: 10.3934/publichealth.2020012
Received: 16 October 2019
Accepted: 03 February 2020
Published: 04 March 2020

http://www.aimspress.com/journal/aimsph

Research article

Nutrient intake, introduction of baby cereals and other complementary foods in the diets of infants and toddlers from birth to 23 months of age

Theresa A Nicklas[1,*], Carol E O'Neil[2] and Victor L Fulgoni III[3]

[1] USDA/ARS Children's Nutrition Research Center, Baylor College of Medicine, Houston, 77030, USA
[2] Louisiana State University Agricultural Center (Emeritus), 143 Kenilworth Parkway, Baton Rouge, LA 70808, USA
[3] Nutrition Impact, LLC, 9725 Drive North, Battle Creek, MI, 49014, USA

* **Correspondence:** Email: tnicklas@bcm.edu; Tel: 7137987087.

Supplementary

Supplemental Table 1. demographics of sample of infants 0–3 months of age participating in the National Health and Nutrition Examination Survey (NHANES) 2001–2012.

	Total population (N = 1,134)*		Baby Cereal Consumption Groups					
			Baby cereal non-consumers (n = 920)*		Non-rice baby cereal consumers (n = 53)*		Rice baby cereal consumers (n = 161)*	
**Variables**	**Mean**	**SE**	**Mean**	**SE**	**Mean**	**SE**	**Mean**	**SE**
Age Months (mean)	1.5	0	1.3[a,c]	0	2.2[b]	0.2	2.0[d]	0.1
Males (%)	53.6	1.9	54.3	2.3	46.9	8.3	51.5	5.5
Race/Ethnicity (%)								
Mexican American	17.5	1.6	19.6[c]	1.8	10.8	5	8.2[d]	2.1
Other Hispanic	6.6	1	6.6	1.1	14.8	5.4	4.3	1.6
Non-Hispanic White	53.5	2.6	53.2	2.8	44.2	8.9	57.4	4.8
Non-Hispanic Black	14	1.4	11.9[c]	1.3	23.3	6.7	22.8[d]	4
Other	8.4	1	8.7	1.1	7	4	7.3	2.2
Poverty Income Ratio (%)								
< 1.35	42.7	1.9	42.5[a]	2.3	67.2[b,e]	8.3	37.1[f]	4.5
1.35 ≤ 1.85	9.1	1.1	10.0[a]	1.2	3.7[b]	2.1	6	1.9
> 1.85	48.2	2	47.5	2.4	29.1[e]	8.5	56.9[f]	4.6
Recumbent Length (cm)	58.9	0.1	58.5[a,c]	0.2	60.3[b]	0.6	60.4[d]	0.3
Weight for Length Percentile	57.4	1	57	1.2	61.8	5.3	58.3	3
Weight for Length z-score	0.2	0	0.2	0	0.4	0.2	0.3	0.1

Continued on next page

Variables	Total population (N = 1,134)*		Baby Cereal Consumption Groups					
			Baby cereal non-consumers (n = 920)*		Non-rice baby cereal consumers (n = 53)*		Rice baby cereal consumers (n = 161)*	
	Mean	SE	Mean	SE	Mean	SE	Mean	SE
Weight Status (%)								
Overweight	11.3	1.1	11.1	1.2	21.5	7.1	9.4	2.5
Overweight or Obese	18.9	1.4	18.7	1.6	30.8	7.9	16.9	3.8
Obese	7.6	1	7.6	1.1	9.3	4.6	7.5	2.5
Infant Feeding Practice (%)								
Breast fed, no formula	29.4	2.1	34.0[a,c]	2.5	1.0[b,e]	0.8	12.3[d,f]	3.7
Formula, not breast fed	54.4	2.3	49.2[a,c]	2.7	87.2[b]	4.6	73.5[d]	4.4
Formula and breast fed	16.1	1.4	16.7	1.6	11.7	4.6	13.8	3.4

Abbreviations: SE: Standard error, PTCL: percentile, WIC: Women, Infants and Children. *Sample sizes for the various variables were slightly smaller due to missing data. [a,b] Means with different superscripts indicate significant differences ($p < 0.01$) between baby cereal non-consumers and non-rice cereal consumers. [c,d] Means with different superscripts indicate significant differences ($p < 0.01$) between baby cereal non-consumers and baby rice cereal consumers. [e,f] Means with different superscripts indicate significant differences ($p < 0.01$) between non-rice cereal consumers and baby rice cereal consumers.

Supplemental Table 2. Demographics of sample of infants 4–6 months of age participating in the National Health and Nutrition Examination Survey (NHANES) 2001–2012.

	Total population (N = 878)*		Baby Cereal Consumption Groups					
			Baby cereal non-consumers (n = 354)*		Non-rice baby cereal consumers (n = 180)*		Rice baby cereal consumers (n = 344)*	
**Variables**	**Mean**	**SE**	**Mean**	**SE**	**Mean**	**SE**	**Mean**	**SE**
Age Months (mean)	5.0	0.0	4.9[a]	0.1	5.3[b,e]	0.1	5.0[f]	0.1
Males (%)	48.5	2.1	43.3	3.8	56.6	4.6	49.9	3.5
Race/Ethnicity (%)								
Mexican American	16.4	1.6	21.2	2.7	13.1	2.5	13.0	1.9
Other Hispanic	8.1	1.1	6.0	1.4	9.2	2.5	9.8	1.9
Non-Hispanic White	54.1	2.8	54.1	4.4	48.9	4.3	56.9	3.6
Non-Hispanic Black	13.8	1.5	9.2[a,c]	1.7	20.9[b]	3.4	15.0[d]	2.0
Other	7.5	1.3	9.5	2.4	7.8	2.3	5.2	1.3
Poverty Income Ratio (%)								
< 1.35	40.7	2.4	40.0	3.5	39.1	4.9	42.4	3.2
1.35 ≤ 1.85	10.4	1.6	12.9	2.8	9.5	2.8	8.2	1.8
> 1.85	48.9	2.8	47.1	4.3	51.4	5.1	49.4	3.6
Recumbent Length (cm)	66.3	0.1	66.0[a]	0.2	66.9[b]	0.2	66.3	0.2
Weight for Length Percentile	62.0	1.2	64.0	1.6	58.1	2.7	61.8	2.1
Weight for Length z-score	0.4	0.0	0.5	0.1	0.3	0.1	0.4	0.1
Weight Status (%)								
Overweight	13.1	1.6	12.9	2.7	12.4	3.6	13.8	2.5
Overweight or Obese	23.0	1.8	22.6	3.2	22.3	4.3	23.7	3.0
Obese	9.8	1.2	9.7	2.0	10.0	3.0	9.9	2.0

Continued on next page

Variables	Total population (N = 878)*		Baby Cereal Consumption Groups: Baby cereal non-consumers (n = 354)*		Non-rice baby cereal consumers (n = 180)*		Rice baby cereal consumers (n = 344)*	
	Mean	SE	Mean	SE	Mean	SE	Mean	SE
Infant Feeding Practice (%)								
Breast fed, no formula	24.3	2.1	36.2[a,c]	3.9	18.7[b]	4.3	14.5[d]	2.6
Formula, not breast fed	63.7	2.5	53.0[a,c]	4.0	69.2[b]	5.1	72.3[d]	3.2
Formula and breast fed	11.8	1.5	10.8	2.2	12.1	3.1	12.6	2.8

Abbreviations: SE: Standard error, PTCL: percentile, WIC: Women, Infants and Children. *Sample sizes for the various variables were slightly smaller due to missing data. [a,b] Means with different superscripts indicate significant differences ($p < 0.01$) between baby cereal non-consumers and non-rice cereal consumers. [c,d] Means with different superscripts indicate significant differences ($p < 0.01$) between baby cereal non-consumers and baby rice cereal consumers. [e,f] Means with different superscripts indicate significant differences ($p < 0.01$) between non-rice cereal consumers and baby rice cereal consumers.

Supplemental Table 3. Demographics of sample of infants 7–11 months of age participating in the National Health and Nutrition Examination Survey (NHANES) 2001–2012.

Variables	Total population (N = 1,429)*		Baby Cereal Consumption Groups: Baby cereal non-consumers (n = 668)*		Non-rice baby cereal consumers (n = 364)*		Rice baby cereal consumers (n = 397)*	
	Mean	SE	Mean	SE	Mean	SE	Mean	SE
Age Months (mean)	8.9	0.0	9.3[a,c]	0.1	8.7[b]	0.1	8.6[d]	0.1
Males (%)	49.3	1.6	47.3	2.3	52.5	3.3	49.3	3.2
Race/Ethnicity (%)								
Mexican American	17.8	1.6	22.8[a,c]	2.0	13.4[b]	2.5	14.3[d]	2.1
Other Hispanic	6.0	0.8	6.7	1.2	7.0	1.4	4.0	0.9
Non-Hispanic White	55.3	2.4	51.9	3.1	58.2	3.9	58.0	3.5
Non-Hispanic Black	12.6	1.2	9.8	1.3	13.3	1.9	16.2	2.5

Continued on next page

	Total population (N = 1,429)*		Baby Cereal Consumption Groups					
			Baby cereal non-consumers (n = 668)*		Non-rice baby cereal consumers (n = 364)*		Rice baby cereal consumers (n = 397)*	
Variables	Mean	SE	Mean	SE	Mean	SE	Mean	SE
Other	8.2	0.9	8.8	1.4	8.1	1.7	7.5	1.7
Poverty Income Ratio (%)								
< 1.35	38.0	1.9	43.6[a]	2.8	31.0[b]	3.3	35.9	3.0
1.35 ≤ 1.85	10.4	1.1	12.1	1.8	8.7	2.2	9.3	2.0
> 1.85	51.6	2.1	44.3[a]	3.0	60.3[b]	3.8	54.8	3.2
Recumbent Length (cm)	72.0	0.1	72.5[c]	0.2	72.0	0.2	71.5[d]	0.2
Weight for Length Percentile	61.2	1.1	62.5	1.5	59.2	2.0	61.1	1.9
Weight for Length z-score	0.4	0.0	0.4	0.1	0.3	0.1	0.4	0.1
Weight Status (%)								
Overweight	15.5	1.4	14.6	1.6	15.0	2.2	17.5	3.0
Overweight or Obese	26.5	1.7	27.8	2.3	23.4	2.4	27.4	3.3
Obese	11.0	1.1	13.2	1.8	8.5	1.7	9.9	1.9
Infant Feeding Practice (%)								
Breast fed, no formula	16.3	1.3	20.4	2.2	13.2	2.0	13.0	2.0
Formula, not breast fed	64.9	1.6	54.2[a,c]	2.7	72.2[b]	2.7	74.3[d]	2.7
Formula and breast fed	6.2	0.8	4.5	0.9	5.8	1.5	9.0	2.0

Abbreviations: SE: Standard error, PTCL: percentile, WIC: Women, Infants and Children. *Sample sizes for the various variables were slightly smaller due to missing data. [a,b] Means with different superscripts indicate significant differences ($p < 0.01$) between baby cereal non-consumers and non-rice cereal consumers. [c,d] Means with different superscripts indicate significant differences ($p < 0.01$) between baby cereal non-consumers and baby rice cereal consumers. [e,f] Means with different superscripts indicate significant differences ($p < 0.01$) between non-rice cereal consumers and baby rice cereal consumers.

Supplemental Table 4. Demographics of sample of infants 12–23 months of age participating in the National Health and Nutrition Examination Survey (NHANES) 2001–2012.

	Total population (N = 2,146)*		Baby Cereal Consumption Groups					
			Baby cereal non-consumers (n = 1,968)*		Non-rice baby cereal Consumers (n = 114)*		Rice baby cereal consumers (n = 64)*	
**Variables**	**Mean**	**SE**	**Mean**	**SE**	**Mean**	**SE**	**Mean**	**SE**
Age Months (mean)	17.5	0.1	17.6[a,c]	0.1	15.9[b]	0.4	15.4[d]	0.5
Males (%)	51.8	1.4	52.0	1.5	48.3	6.6	54.4	6.9
Race/Ethnicity (%)								
Mexican American	17.0	1.4	16.9	1.4	13.3	3.6	29.3	7.0
Other Hispanic	8.1	0.9	8.0	0.9	10.4	2.7	7.7	3.1
Non-Hispanic White	55.0	2.0	55.4[c]	2.0	61.3[e]	5.9	21.5[d,f]	6.4
Non-Hispanic Black	12.9	1.0	12.8	1.0	9.3[e]	2.5	28.5[f]	6.4
Other	7.0	0.8	7.0	0.8	5.7	2.3	12.9	4.9
Poverty Income Ratio (%)								
< 1.35	39.7	1.4	40.2	1.5	28.9	5.9	51.0	7.0
1.35 ≤ 1.85	10.3	1.0	10.2	1.1	11.0	4.8	14.4	6.3
> 1.85	49.9	1.7	49.6	1.7	60.1	6.8	34.6	7.4
Recumbent Length (cm)	81.6	0.1	81.8[c]	0.1	80.1	0.7	79.5[d]	0.7
Weight for Length Percentile	55.4	1.0	55.3	1.0	59.3	3.5	50.9	4.2
Weight for Length z-score	0.2	0.0	0.2	0.0	0.3	0.1	0.1	0.1
Weight Status (%)								
Overweight	12.5	1.2	12.3	1.2	16.4	5.7	10.4	4.5
Overweight or Obese	20.2	1.3	20.0	1.4	25.3	5.8	18.2	5.4
Obese	7.8	0.7	7.7	0.7	8.9	2.9	7.7	3.2

Continued on next page

Variables	Total population (N = 2,146)*		Baby Cereal Consumption Groups: Baby cereal non-consumers (n = 1,968)*		Non-rice baby cereal Consumers (n = 114)*		Rice baby cereal consumers (n = 64)*	
	Mean	SE	Mean	SE	Mean	SE	Mean	SE
Infant Feeding Practice (%)								
Breast fed, no formula	6.6	0.8	6.5	0.8	6.8	3.3	8.0	3.4
Formula, not breast fed	5.6	0.7	4.2[a,c]	0.7	19.0[b]	4.8	31.0 [d]	7.4
Formula and breast fed	0.1	0.1	0.1	0.1	0.0	0.0	1.1	1.1

Abbreviations: SE: Standard error, PTCL: percentile, WIC: Women, Infants and Children. *Sample sizes for the various variables were slightly smaller due to missing data. [a,b] Means with different superscripts indicate significant differences ($p < 0.01$) between baby cereal non-consumers and non-rice cereal consumers. [c,d] Means with different superscripts indicate significant differences ($p < 0.01$) between baby cereal non-consumers and baby rice cereal consumers. [e,f] Means with different superscripts indicate significant differences ($p < 0.01$) between non-rice cereal consumers and baby rice cereal consumers.

Supplemental Table 5. Introduction of foods for 0–3 months of age by baby cereal consumption groups.

Main Groups	Subgroups	Baby Cereal Consumption Groups: Baby cereal non-consumers		Non-rice baby cereal consumers		Rice baby cereal consumers	
0-3 months		Mean	SE	Mean	SE	Mean	SE
**Infant Formula and Baby Foods**		523.72[a,c]	25.47	856.40[b]	41.44	801.6[d]	46.26
	Infant Formulas	518.12[a,c]	25.10	812.82[b]	42.09	767.81[d]	45.85
	Baby Beverages	3.45	0.80	21.87	8.02	5.52	1.65
	Baby Foods	2.15[a,c]	0.63	21.71[b]	3.85	28.29[d]	3.67
**Water**		4.62[c]	0.87	44.57	27.62	14.88[d]	3.68
	Plain Water	4.62[c]	0.87	44.57	27.62	14.88[d]	3.68
**Beverages, Nonalcoholic**		2.74	1.12	12.93	5.12	1.95	0.88
	Sweetened Beverages	2.39	1.09	7.03	3.96	0.70	0.51
	Coffee and Tea	0.23	0.11	4.02	3.00	0.00	0.00
	100% Juice	0.12	0.04	1.88	1.85	1.25	0.72

Continued on next page

Main Groups	Subgroups	Baby Cereal Consumption Groups					
		Baby cereal non-consumers		Non-rice baby cereal consumers		Rice baby cereal consumers	
**0-3 months**		**Mean**	**SE**	**Mean**	**SE**	**Mean**	**SE**
**Vegetables**		0.48	0.28	2.52	2.08	0.13	0.11
	White Potatoes	0.28	0.26	2.52	2.08	0.11	0.11
	Vegetables, excluding Potatoes	0.20	0.12	0.00	0.00	0.02	0.02
**Fruit**		0.34	0.23	2.88	2.44	1.37	1.23
	Fruits	0.34	0.23	2.88	2.44	1.37	1.23
**Sugars**		0.10	0.08	0.09	0.09	0.07	0.05
	Sugars	0.10	0.08	0.09	0.09	0.07	0.05
**Condiments and Sauces**		0.06	0.06	0.00	0.00	0.00	0.00
	Condiments and Sauces	0.06	0.06	0.00	0.00	0.00	0.00
**Milk and Dairy**		0.06	0.04	0.00	.*	2.84	2.80
	Milk	0.04	0.04	0.00	0.00	2.84	2.80
	Yogurt	0.02	0.02	0.00	0.00	0.00	0.00
**Snacks and Sweets**		0.02	0.01	0.00	0.00	0.00	0.00
	Other Desserts	0.02	0.01	0.00	0.00	0.00	0.00
**Mixed Dishes**		0.01	0.01	0.00	0.00	0.12	0.12
	Mixed Dishes - Soups	0.01	0.01	0.00	0.00	0.12	0.12
**Grains**		0.00	0.00	0.02	0.02	0.00	0.00
	Breads, Rolls, Tortillas	0.00	0.00	0.02	0.02	0.00	0.00

Abbreviations: SE: Standard error. *All subjects have zero consumption and thus there are no variance estimates. [a,b] Means with different superscripts indicate significant differences ($p < 0.01$) between baby cereal non-consumers and non-rice cereal consumers. [c,d] Means with different superscripts indicate significant differences ($p < 0.01$) between baby cereal non-consumers and baby rice cereal consumers. [e,f] Means with different superscripts indicate significant differences ($p < 0.01$) between non-rice cereal consumers and baby rice cereal consumers.

Supplemental Table 6. Introduction of foods for 4–6 months of age by baby cereal consumption groups.

Main Groups	Subgroups	Baby Cereal Consumption Groups					
		Baby cereal non-consumers		Non-rice baby cereal consumers		Rice baby cereal consumers	
**4–6 months**		**Mean**	**SE**	**Mean**	**SE**	**Mean**	**SE**
**Infant Formula and Baby Foods**		650.76[c]	46.26	793.40	51.72	901.33[d]	34.31
	Infant Formulas	573.04[c]	41.33	618.46	48.27	745.41[d]	30.28
	Baby Foods	60.57[a,c]	7.60	144.26[b]	13.10	129.99[d]	8.25
	Baby Beverages	17.15	5.13	30.67	5.92	25.93	3.40
**Water**		11.54[a,c]	2.34	29.60[b]	4.67	24.24[d]	3.16
	Plain Water	11.54[a,c]	2.34	29.60[b]	4.67	23.23[d]	2.77
	Flavored or Enhanced Water	0.00	0.00	0.00	0.00	1.01	1.00
**Beverages, Nonalcoholic**		10.73	3.03	17.69	7.29	14.77	6.53
	Sweetened Beverages	5.38	2.52	7.44	6.61	7.67	6.20
	100% Juice	3.74	1.31	9.92	3.15	6.61	1.40
	Coffee and Tea	1.61	1.03	0.32	0.19	0.50	0.50
**Vegetables**		4.77	1.65	13.31	4.45	3.16	0.80
	Vegetables, excluding Potatoes	3.88	1.65	10.06	4.46	2.19	0.60
	White Potatoes	0.89	0.33	3.25	0.89	0.96	0.46
**Fruit**		4.73	1.53	5.39	2.02	4.41	1.41
	Fruits	4.73	1.53	5.39	2.02	4.41	1.41

Continued on next page

Main Groups	Subgroups	Baby Cereal Consumption Groups					
		Baby cereal non-consumers		Non-rice baby cereal consumers		Rice baby cereal consumers	
**4–6 months**		**Mean**	**SE**	**Mean**	**SE**	**Mean**	**SE**
**Mixed Dishes**		4.25	1.08	3.96	1.23	4.21	1.31
	Mixed Dishes - Soups	3.75	1.03	2.00	0.74	2.50	0.92
	Mixed Dishes - Grain-based	0.50	0.27	1.66	1.01	0.80	0.48
	Mixed Dishes - Meat, Poultry, Seafood	0.00	0.00	0.17	0.17	0.83	0.82
	Mixed Dishes - Asian	0.00	0.00	0.03	0.02	0.05	0.04
	Mixed Dishes - Sandwiches (single code)	0.00	0.00	0.00	0.00	0.05	0.05
	Mixed Dishes - Pizza	0.00	0.00	0.11	0.11	0.00	0.00
**Milk and Dairy**		3.41	1.65	13.53	9.83	3.48	2.48
	Yogurt	2.42	1.41	1.67	1.16	0.03	0.03
	Milk	0.95	0.86	1.43	1.10	3.42	2.48
	Cheese	0.04	0.03	0.04	0.02	0.03	0.03
	Dairy Drinks and Substitutes	0.00	0.00	9.76	9.71	0.00	0.00
	Flavored Milk	0.00	0.00	0.62	0.63	0.00	0.00
**Protein Foods**		1.23	0.45	1.16	0.43	0.48	0.17
	Eggs	0.57	0.20	0.57	0.29	0.11	0.07
	Plant-based Protein Foods	0.53	0.28	0.12	0.09	0.19	0.11
	Poultry	0.08	0.05	0.36	0.24	0.17	0.07
	Cured Meats/Poultry	0.04	0.01	0.11	0.09	0.01	0.01
	Seafood	0.00	0.00	0.00	0.00	0.01	0.01
**Snacks and Sweets**		0.87	0.30	2.94	1.05	1.12	0.41
	Other Desserts	0.36	0.26	1.60	0.83	0.31	0.26
	Sweet Bakery Products	0.35	0.13	0.67	0.31	0.70	0.32
	Crackers	0.14	0.05	0.50	0.32	0.10	0.04
	Savory Snacks	0.01	0.01	0.02	0.01	0.02	0.01
	Candy	0.00	0.00	0.15	0.12	0.00	0.00

Continued on next page

Main Groups	Subgroups	Baby Cereal Consumption Groups					
		Baby cereal non-consumers		Non-rice baby cereal consumers		Rice baby cereal consumers	
**4–6 months**		**Mean**	**SE**	**Mean**	**SE**	**Mean**	**SE**
**Grains**		0.86	0.27	5.95	3.74	2.42	0.91
	Cooked Grains	0.43	0.21	0.88	0.83	0.33	0.25
	Breads, Rolls, Tortillas	0.34	0.16	0.91	0.47	0.22	0.11
	Cooked Cereals	0.06	0.06	4.00	3.54	1.83	0.93
	Ready-to-Eat Cereals	0.02	0.02	0.15	0.11	0.03	0.02
	Quick Breads and Bread Products	0.01	0.01	0.00	.*	0.01	0.01
**Sugars**		0.12	0.11	0.04	0.02	0.04	0.02
**Fats and Oils**		0.07	0.04	0.00	0.00	0.01	0.01
	Fats and Oils	0.07	0.04	0.00	0.00	0.01	0.01
**Condiments and Sauces**		0.00	0.00	0.03	0.02	0.00	0.00
	Condiments and Sauces	0.00	0.00	0.03	0.02	0.00	0.00

Abbreviations: SE: Standard error. *All subjects have zero consumption and thus there are no variance estimates. [a,b] Means with different superscripts indicate significant differences ($p < 0.01$) between baby cereal non-consumers and non-rice cereal consumers. [c,d] Means with different superscripts indicate significant differences ($p < 0.01$) between baby cereal non-consumers and baby rice cereal consumers. [e,f] Means with different superscripts indicate significant differences ($p < 0.01$) between non-rice cereal consumers and baby rice cereal consumers.

Supplemental Table 7. Introduction of foods for 7–11 months of age by baby cereal consumption groups.

Main Groups	Subgroups	Baby Cereal Consumption Groups					
		Baby cereal non-consumers		Non-rice baby cereal consumers		Rice baby cereal consumers	
**7–11 months**		**Mean**	**SE**	**Mean**	**SE**	**Mean**	**SE**
**Infant Formula and Baby Foods**		571.74[a,c]	27.84	886.19[b]	31.96	887.69[d]	28.09
	Infant Formulas	453.30[a,c]	25.05	604.19[b]	27.70	623.34[d]	24.21
	Baby Foods	85.91[a,c]	6.99	234.54[b]	14.28	218.30[d]	11.93
	Baby Beverages	32.54	4.27	47.46	5.39	46.04	5.54
**Milk and Dairy**		189.85[a,c]	14.73	69.07[b]	10.66	47.24[d]	8.42
	Milk	165.07[a,c]	13.69	55.64[b]	10.09	37.88[d]	7.99
	Yogurt	15.29[a,c]	2.80	6.92[b]	1.44	4.46[d]	1.76
	Dairy Drinks and Substitutes	3.99	2.05	1.45	1.44	2.03	1.73
	Cheese	3.68	0.54	1.83	0.63	2.28	0.73
	Flavored Milk	1.81	0.72	3.23	1.56	0.60	0.60
**Water**		79.44	7.99	93.61	17.70	79.00	10.88
	Plain Water	79.38	7.99	93.61	17.70	77.86	10.87
	Flavored or Enhanced Water	0.05	0.05	0.00	0.00	1.15	1.14
**Beverages, Nonalcoholic**		75.54[a]	6.62	32.99[b]	6.38	51.42	6.63
	100% Juice	47.63[a]	5.18	24.24[b]	5.78	34.49	5.53
	Sweetened Beverages	24.55[a]	4.17	4.73[b]	1.23	14.71	4.48
	Coffee and Tea	3.29	1.16	2.68	1.22	1.87	0.90
	Diet Beverages	0.07	0.05	1.34	1.32	0.35	0.27

Continued on next page

Main Groups	Subgroups	Baby Cereal Consumption Groups					
		Baby cereal non-consumers		Non-rice baby cereal consumers		Rice baby cereal consumers	
7–11 months		Mean	SE	Mean	SE	Mean	SE
**Mixed Dishes**		64.94[a,c]	4.97	29.49[b]	4.32	26.54[d]	3.75
	Mixed Dishes - Grain-based	31.06[a,c]	3.70	11.98[b]	2.31	12.15[d]	2.98
	Mixed Dishes - Soups	21.10[c]	3.09	11.77	2.69	10.71[d]	2.03
	Mixed Dishes - Meat, Poultry, Seafood	7.48[c]	1.49	5.00	1.08	2.88[d]	0.76
	Mixed Dishes - Sandwiches (single code)	1.50	0.59	0.19	0.13	0.00	0.00
	Mixed Dishes - Mexican	1.46	0.60	0.26	0.14	0.00	0.00
	Mixed Dishes - Pizza	1.42[a]	0.43	0.28[b]	0.09	0.27	0.19
	Mixed Dishes - Asian	0.91	0.42	0.02	0.02	0.52	0.26
**Fruit**		50.46[a,c]	4.29	31.4[b]	4.66	22.40[d]	3.18
	Fruits	50.46[a,c]	4.29	31.4[b]	4.66	22.40[d]	3.18
**Protein Foods**		33.41[a,c]	4.24	10.02[b]	1.75	9.80[d]	1.70
	Eggs	10.57[a,c]	1.43	3.21[b]	1.17	2.01[d]	0.55
	Plant-based Protein Foods	7.46	2.23	2.49	0.66	2.71	1.21
	Poultry	7.43[a,c]	1.00	1.50[b]	0.37	2.55[d]	0.60
	Cured Meats/Poultry	5.89[a,c]	1.51	1.74[b]	0.51	1.48[d]	0.40
	Meats	1.56	0.40	1.01	0.36	0.74	0.39
	Seafood	0.51	0.20	0.08	0.05	0.31	0.20
**Vegetables**		32.11[a,c]	3.84	15.61[b]	2.03	15.73[d]	3.10
	Vegetables, excluding Potatoes	22.24[a,c]	3.26	10.78[b]	1.71	9.63[d]	2.83
	White Potatoes	9.87[a]	1.45	4.83[b]	1.15	6.10	1.19

Continued on next page

Main Groups	Subgroups	Baby Cereal Consumption Groups					
		Baby cereal non-consumers		Non-rice baby cereal consumers		Rice baby cereal consumers	
7–11 months		Mean	SE	Mean	SE	Mean	SE
**Grains**		28.97[a,c]	2.79	13.04[b]	1.89	11.37[d]	2.17
	Cooked Cereals	10.19[a,c]	2.39	2.27[b]	1.00	2.14[d]	0.75
	Breads, Rolls, Tortillas	7.67[a,c]	0.75	2.93[b]	0.70	3.15[d]	0.59
	Cooked Grains	4.20	0.95	4.77	1.08	3.57	1.42
	Quick Breads and Bread Products	3.83[c]	0.70	1.69	0.91	1.10[d]	0.45
	Ready-to-Eat Cereals	3.07[a,c]	0.38	1.38[b]	0.26	1.41[d]	0.32
**Snacks and Sweets**		16.35[a,c]	1.39	6.15[b]	0.97	8.60[d]	1.02
	Sweet Bakery Products	6.86[a,c]	0.84	2.19[b]	0.49	2.13[d]	0.34
	Other Desserts	4.24[a]	0.63	1.30[b]	0.63	3.19	0.82
	Crackers	2.19[a]	0.30	1.20[b]	0.23	2.16	0.37
	Savory Snacks	1.58	0.35	0.82	0.20	0.87	0.18
	Snack/Meal Bars	0.76	0.38	0.21	0.16	0.01	0.01
	Candy	0.72	0.29	0.43	0.20	0.24	0.16
**Condiments and Sauces**		2.14[a,c]	0.66	0.31[b]	0.14	0.30[d]	0.10
	Condiments and Sauces	2.14[a,c]	0.66	0.31[b]	0.14	0.30[d]	0.10
**Fats and Oils**		0.58[a]	0.12	0.09[b]	0.04	0.30	0.10
	Fats and Oils	0.58[a]	0.12	0.09[b]	0.04	0.30	0.10
**Sugars**		0.45	0.11	0.19	0.08	0.23	0.11
	Sugars	0.45	0.11	0.19	0.08	0.23	0.11
**Other**		0.08	0.04	0.03	0.03	0.00	0.00
	Other	0.08	0.04	0.03	0.03	0.00	0.00

Abbreviations: SE: Standard error. [a,b] Means with different superscripts indicate significant differences ($p < 0.01$) between baby cereal non-consumers and non-rice cereal consumers. [c,d] Means with different superscripts indicate significant differences ($p < 0.01$) between baby cereal non-consumers and baby rice cereal consumers. [e,f] Means with different superscripts indicate significant differences ($p < 0.01$) between non-rice cereal consumers and baby rice cereal consumers.

Supplemental Table 8. Introduction of foods for 12–23 months of age by baby cereal consumption groups.

Main Groups	Subgroups	Baby Cereal Consumption Groups					
		Baby cereal non-consumers		Non-rice baby cereal consumers		Rice baby cereal consumers	
12–23 months		Mean	SE	Mean	SE	Mean	SE
**Milk and Dairy**		544.42	11.82	478.97	38.86	534.29	67.82
	Milk	475.66	12.43	394.92	39.03	452.75	59.75
	Yogurt	22.14	1.80	22.35	7.80	40.14	14.54
	Flavored Milk	18.91	3.03	8.85	7.04	36.70	32.00
	Dairy Drinks and Substitutes	16.49[c]	2.74	46.79	24.65	0.00[d]	0.00
	Cheese	11.22[a,c]	0.92	6.07[b]	1.43	4.70[d]	1.10
**Beverages, Nonalcoholic**		285.37[a,c]	10.58	191.44[b]	30.08	195.53[d]	32.06
	100% Juice	155.67	7.72	114.55	19.35	125.98	28.49
	Sweetened Beverages	108.43	6.97	63.66	26.55	66.34	24.10
	Coffee and Tea	16.42[c]	3.43	7.09	3.73	1.52[d]	1.50
	Diet Beverages	4.85	1.31	6.15	5.23	1.69	1.26
**Water**		184.53[c]	7.72	173.69	24.49	112.66[d]	25.41
	Plain Water	182.27[c]	7.77	173.69	24.49	110.49[d]	24.91
	Flavored or Enhanced Water	2.26	1.11	0.00	0.00	2.17	2.12
**Mixed Dishes**		122.03[a]	4.45	87.27[b]	11.24	104.69	15.30
	Mixed Dishes - Grain-based	47.57[a]	2.58	27.85[b]	6.99	34.48	9.83
	Mixed Dishes - Soups	31.98	3.91	23.18	7.86	41.32	12.02
	Mixed Dishes - Meat, Poultry, Seafood	18.22	1.87	30.05	8.52	22.32	11.50
	Mixed Dishes - Pizza	8.29[a,c]	0.73	2.48[b]	1.40	1.92[d]	1.12
	Mixed Dishes - Mexican	6.72[a]	1.07	2.11[b]	1.04	2.45	2.19
	Mixed Dishes - Sandwiches (single code)	6.38[a,c]	0.78	0.49[b]	0.38	0.00[d]	0.00
	Mixed Dishes - Asian	2.88	0.66	1.10	0.60	2.20	1.43

Continued on next page

Main Groups	Subgroups	Baby Cereal Consumption Groups					
		Baby cereal non-consumers		Non-rice baby cereal consumers		Rice baby cereal consumers	
12–23 months		Mean	SE	Mean	SE	Mean	SE
**Fruit**		90.48	3.01	97.55	14.46	75.85	10.55
	Fruits	90.48	3.01	97.55	14.46	75.85	10.55
**Grains**		64.88[c]	2.44	48.52	7.00	33.99[d]	6.31
	Cooked Cereals	19.99	1.86	12.34	4.79	11.98	5.00
	Breads, Rolls, Tortillas	16.70[c]	0.77	17.13	4.20	7.95[d]	2.09
	Cooked Grains	12.51	1.12	10.52	3.52	6.26	2.44
	Ready-to-Eat Cereals	8.66	0.46	6.41	1.64	3.89	1.17
	Quick Breads and Bread Products	7.02[a]	0.69	2.12[b]	0.93	3.92	2.26
**Protein Foods**		64.40[a,c]	1.94	33.95[b]	7.37	38.28[d]	7.05
	Poultry	18.17	1.06	13.74	5.19	10.64	3.56
	Eggs	17.30[a]	1.18	6.60[b]	2.22	11.58	3.52
	Cured Meats/Poultry	12.45[a,c]	0.86	4.82[b]	1.75	4.10[d]	1.56
	Plant-based Protein Foods	9.22	0.89	5.27	2.20	5.44	2.26
	Meats	5.27	0.49	2.85	1.39	4.52	2.75
	Seafood	1.99	0.34	0.66	0.50	2.01	1.79
**Infant Formula and Baby Foods**		51.36[a,c]	4.72	323.67[b]	50.15	354.57[d]	73.79
	Infant Formulas	21.88[a,c]	3.85	112.83[b]	29.17	218.20[d]	65.07
	Baby Foods	16.20[a,c]	2.09	176.72[b]	27.87	86.82[d]	18.64
	Baby Beverages	13.28	2.07	34.11	13.67	49.55	16.92

Continued on next page

Main Groups	Subgroups	Baby Cereal Consumption Groups					
		Baby cereal non-consumers		Non-rice baby cereal consumers		Rice baby cereal consumers	
12–23 months		Mean	SE	Mean	SE	Mean	SE
**Snacks and Sweets**		47.99[a,c]	1.51	27.70[b]	4.02	24.53[d]	4.35
	Sweet Bakery Products	15.55	0.81	11.88	2.07	11.40	3.17
	Other Desserts	12.94[a,c]	1.08	3.65[b]	1.45	3.39[d]	1.78
	Crackers	6.86	0.42	3.95	1.06	4.09	1.74
	Savory Snacks	6.24[c]	0.37	4.42	1.85	1.52[d]	0.46
	Candy	4.74	0.62	2.24	0.91	3.66	1.75
	Snack/Meal Bars	1.65	0.26	1.56	1.25	0.48	0.48
**Vegetables**		47.60[c]	1.76	37.11	8.76	14.77[d]	3.88
	Vegetables, excluding Potatoes	29.44[c]	1.46	20.48	4.29	11.21[d]	3.31
	White Potatoes	18.16[c]	1.09	16.62	7.73	3.56[d]	1.75
**Condiments and Sauces**		5.02[c]	0.60	2.68	1.89	0.57[d]	0.26
	Condiments and Sauces	5.02[c]	0.60	2.68	1.89	0.57[d]	0.26
**Sugars**		2.48[c]	0.20	1.34	0.53	1.28[d]	0.44
	Sugars	2.48[c]	0.20	1.34	0.53	1.28[d]	0.44
**Fats and Oils**		1.81[a,c]	0.18	0.83[b]	0.32	0.68[d]	0.27
	Fats and Oils	1.81[a,c]	0.18	0.83[b]	0.32	0.68[d]	0.27
**Other**		0.48[a]	0.13	0.04[b]	0.03	1.71	1.61
	Other	0.34[a]	0.11	0.04[b]	0.03	1.71	1.61
	Protein and Nutritional Powders	0.14	0.08	0.00	0.00	0.00	0.00

Abbreviations: SE: Standard error. [a,b] Means with different superscripts indicate significant differences ($p < 0.01$) between baby cereal non-consumers and non-rice cereal consumers. [c,d] Means with different superscripts indicate significant differences ($p < 0.01$) between baby cereal non-consumers and baby rice cereal consumers. [e,f] Means with different superscripts indicate significant differences ($p < 0.01$) between non-rice cereal consumers and baby rice cereal consumers.

Supplemental Table 9. Introduction of foods for 0-3 months of age by baby cereal consumption groups.

Subgroups	Food Categories	Baby Cereal Consumption Groups					
		Baby cereal non-consumers		Non-rice baby cereal consumers		Rice baby cereal consumers	
0–3 months		Mean	SE	Mean	SE	Mean	SE
Infant Formulas							
	Formula, prepared from powder	454.23	25.27	603.31	58.94	578.23	49.05
	Formula, prepared from concentrate	46.31	6.62	181.88	60.93	107.29	26.08
	Formula, ready-to-feed	17.58	3.67	27.63	25.81	82.28	31.83
Baby Beverages							
	Baby juice	2.04	0.69	15.07	7.49	3.39	1.29
	Baby water	1.41	0.38	6.80	3.68	2.13	1.07
Baby Foods							
	Baby food: fruit	0.90	0.34	7.45	2.54	4.55	1.56
	Baby food: vegetable	0.89	0.29	1.60	1.01	6.26	2.03
	Baby food: meat and dinners	0.20	0.20	0.00	0.00	0.00	0.00
	Baby food: cereals	0.07[a,c]	0.07	11.94[b]	2.23	17.47[d]	1.55
	Baby food: yogurt	0.06	0.06	0.66	0.65	0.00	0.00
	Baby food: snacks and sweets	0.04	0.04	0.07	0.08	0.01	0.01
Plain Water							
	Bottled water	2.54	0.61	3.47	2.26	3.06	1.69
	Tap water	2.08[c]	0.67	41.09	27.71	11.83[d]	3.39
Sweetened Beverages							
	Sport and energy drinks	2.24	1.08	3.92	2.54	0.70	0.51
	Fruit drinks	0.12	0.12	3.11	3.09	0.00	0.00
	Soft drinks	0.03	0.02	0.00	0.00	0.00	0.00

Continued on next page

Subgroups	Food Categories	Baby Cereal Consumption Groups					
		Baby cereal non-consumers		Non-rice baby cereal consumers		Rice baby cereal consumers	
0–3 months		Mean	SE	Mean	SE	Mean	SE
Coffee and Tea							
	Tea	0.23	0.11	4.02	3.00	0.00	0.00
100% Juice							
	Other fruit juice	0.08	0.05	1.88	1.85	0.55	0.55
White Potatoes	French fries and other fried white potatoes	0.00	0.00	0.00	0.00	0.00	0.00
Vegetables, excluding Potatoes							
	Other starchy vegetables	0.09	0.09	0.00	.*	0.00	0.00
	Other red and orange vegetables	0.07	0.07	0.00	.*	0.00	0.00
	Carrots	0.03	0.03	0.00	0.00	0.02	0.02
	Other vegetables and combinations	0.01	0.01	0.00	0.00	0.00	0.00
Fruits							
	Apples	0.17	0.12	2.46	2.41	1.26	1.22
	Bananas	0.16	0.13	0.42	0.43	0.11	0.11
Sugars							
	Jams, syrups, toppings	0.08	0.08	0.09	0.09	0.04	0.04
	Sugars and honey	0.02	0.01	0.01	0.01	0.03	0.03
Condiments and Sauces							
	Dips, gravies, other sauces	0.06	0.06	0.00	0.00	0.00	0.00
Milk							
	Milk, whole	0.04	0.04	0.00	0.00	2.84	2.80
Yogurt							
	Yogurt, regular	0.02	0.02	0.00	0.00	0.00	0.00

Continued on next page

Subgroups	Food Categories	Baby Cereal Consumption Groups					
		Baby cereal non-consumers		Non-rice baby cereal consumers		Rice baby cereal consumers	
0–3 months		Mean	SE	Mean	SE	Mean	SE
Other Desserts							
	Gelatins, ices, sorbets	0.01	0.01	0.00	.*	0.00	0.00
	Ice cream and frozen dairy desserts	0.01	0.00	0.00	0.00	0.00	0.00
Mixed Dishes - Soups							
	Soups	0.01	0.01	0.00	0.00	0.12	0.12
Breads, Rolls, Tortillas							
	Yeast breads	0.00	0.00	0.02	0.02	0.00	0.00

Abbreviations: SE: Standard error. *All subjects have zero consumption and thus there are no variance estimates. [a,b] Means with different superscripts indicate significant differences ($p < 0.01$) between baby cereal non-consumers and non-rice cereal consumers. [c,d] Means with different superscripts indicate significant differences ($p < 0.01$) between baby cereal non-consumers and baby rice cereal consumers. [e,f] Means with different superscripts indicate significant differences ($p < 0.01$) between non-rice cereal consumers and baby rice cereal consumers.

Supplemental Table 10. Introduction of foods for 4–6 months of age by baby cereal consumption groups.

Subgroups	Food Categories	Baby Cereal Consumption Groups					
		Baby cereal non-consumers		Non-rice baby cereal consumers		Rice baby cereal consumers	
4–6 months		Mean	SE	Mean	SE	Mean	SE
Infant Formulas							
	Formula, prepared from powder	477.81	38.05	504.08	49.50	611.03	34.55
	Formula, prepared from concentrate	70.34	21.40	77.02	28.64	84.51	19.52
	Formula, ready-to-feed	24.90	9.75	37.37	12.69	49.88	13.68

Continued on next page

Subgroups	Food Categories	Baby Cereal Consumption Groups					
		Baby cereal non-consumers		Non-rice baby cereal consumers		Rice baby cereal consumers	
4–6 months		Mean	SE	Mean	SE	Mean	SE
Baby Foods							
	Baby food: fruit	25.05[a,c]	3.63	48.15[b]	6.48	46.67[d]	5.03
	Baby food: vegetable	23.62[c]	3.99	40.19	5.42	41.62[d]	3.88
	Baby food: meat and dinners	7.74	2.32	16.46	3.50	10.62	1.74
	Baby food: snacks and sweets	2.90	1.25	6.73	2.72	3.36	0.94
	Baby food: yogurt	1.26	0.60	1.47	0.90	1.22	0.69
	Baby food: cereals	0.00[a,c]	0.00	31.27[b]	3.61	26.50[d]	2.46
Baby Beverages							
	Baby juice	14.89	5.11	24.68	5.18	20.21	3.02
	Baby water	2.26	0.68	5.99	2.02	5.71	1.36
Plain Water							
	Tap water	7.58[a,c]	1.88	21.48[b]	4.25	17.85[d]	2.52
	Bottled water	3.96	1.20	8.11	1.91	5.38	1.41
Flavored or Enhanced Water							
	Enhanced or fortified water	0.00	0.00	0.00	0.00	1.01	1.00
Sweetened Beverages							
	Sport and energy drinks	4.87	2.48	0.69	0.44	6.21	6.11
	Smoothies and grain drinks	0.41	0.41	0.00	0.00	0.15	0.15
	Fruit drinks	0.10	0.07	6.75	6.59	1.25	1.19
	Soft drinks	0.01	0.01	0.00	0.00	0.06	0.06
100% Juice							
	Apple juice	3.13	1.27	3.65	1.44	3.45	1.13
	Other fruit juice	0.32	0.19	5.77	2.41	2.89	1.11
	Citrus juice	0.28	0.20	0.50	0.50	0.01	0.01

Continued on next page

Subgroups	Food Categories	Baby Cereal Consumption Groups					
		Baby cereal non-consumers		Non-rice baby cereal consumers		Rice baby cereal consumers	
**4–6 months**		**Mean**	**SE**	**Mean**	**SE**	**Mean**	**SE**
Coffee and Tea							
	Tea	1.61	1.03	0.32	0.19	0.50	0.50
Vegetables, excluding Potatoes							
	Other red and orange vegetables	1.67	1.00	6.92	4.16	0.66	0.35
	Other vegetables and combinations	1.09	0.94	2.59	1.49	0.82	0.35
	Carrots	0.51	0.32	0.39	0.37	0.57	0.19
	Tomatoes	0.14	0.12	0.00	0.00	0.01	0.01
	Dark green vegetables, excludes lettuce	0.09	0.07	0.07	0.07	0.08	0.06
	String beans	0.03	0.03	0.01	0.01	0.04	0.04
	Corn	0.01	0.01	0.00	0.00	0.00	0.00
White Potatoes							
	Mashed potatoes and white potato mixtures	0.67	0.30	2.75	0.92	0.63	0.41
	White potatoes, baked or boiled	0.18	0.13	0.24	0.24	0.23	0.14
	French fries and other fried white potatoes	0.04	0.02	0.26	0.16	0.11	0.08

Continued on next page

Subgroups	Food Categories	Baby Cereal Consumption Groups					
		Baby cereal non-consumers		Non-rice baby cereal consumers		Rice baby cereal consumers	
**4–6 months**		**Mean**	**SE**	**Mean**	**SE**	**Mean**	**SE**
Fruits							
	Bananas	2.29	0.84	2.23	0.97	1.02	0.37
	Apples	1.73	0.81	0.86	0.41	1.70	1.17
	Other fruits and fruit salads	0.52	0.42	1.59	1.10	1.23	0.81
	Citrus fruits	0.13	0.09	0.08	0.08	0.02	0.02
	Melons	0.04	0.04	0.00	.	0.17	0.16
	Peaches and nectarines	0.03	0.03	0.01	0.01	0.00	0.00
	Berries	0.00	0.00	0.08	0.08	0.00	0.00
	Dried fruits	0.00	0.00	0.00	0.00	0.24	0.21
	Grapes	0.00	0.00	0.53	0.53	0.04	0.04
Mixed Dishes - Soups							
	Soups	3.75	1.03	2.00	0.74	2.50	0.92
Mixed Dishes - Grain-based							
	Rice mixed dishes	0.28	0.16	0.47	0.36	0.14	0.11
	Macaroni and cheese	0.22	0.22	0.00	0.00	0.00	0.00
Mixed Dishes - Meat, Poultry, Seafood							
	Poultry mixed dishes	0.00	0.00	0.17	0.17	0.00	0.00
	Meat mixed dishes	0.00	0.00	0.00	0.00	0.83	0.82
Mixed Dishes - Asian							
	Fried rice and lo/chow mein	0.00	0.00	0.01	0.01	0.01	0.01
	Stir-fry and soy-based sauce mixtures	0.00	0.00	0.02	0.02	0.03	0.03

Continued on next page

Subgroups	Food Categories	Baby Cereal Consumption Groups					
		Baby cereal non-consumers		Non-rice baby cereal consumers		Rice baby cereal consumers	
**4–6 months**		**Mean**	**SE**	**Mean**	**SE**	**Mean**	**SE**
Mixed Dishes - Sandwiches (single code)							
	Egg/breakfast sandwiches (single code)	0.00	0.00	0.00	0.00	0.05	0.05
Mixed Dishes - Pizza							
	Pizza	0.00	0.00	0.11	0.11	0.00	0.00
Yogurt							
	Yogurt, regular	2.42	1.41	1.58	1.15	0.03	0.03
	Yogurt, Greek	0.00	0.00	0.10	0.09	0.00	0.00
Milk							
	Milk, whole	0.95	0.86	0.69	0.69	3.26	2.48
	Milk, lowfat	0.00	0.00	0.00	0.00	0.06	0.06
	Milk, reduced fat	0.00	0.00	0.34	0.34	0.08	0.08
	Milk, nonfat	0.00	0.00	0.39	0.40	0.02	0.02
Cheese							
	Cheese	0.04	0.03	0.04	0.02	0.03	0.03
Dairy Drinks and Substitutes							
	Milk shakes and other dairy drinks	0.00	0.00	0.00	0.00	0.00	0.00
	Milk substitutes	0.00	0.00	9.76	9.71	0.00	0.00
Flavored Milk							
	Flavored milk, lowfat	0.00	0.00	0.62	0.63	0.00	0.00
Eggs							
	Eggs and omelets	0.57	0.20	0.57	0.29	0.11	0.07
Plant-based Protein Foods							
	Nuts and seeds	0.00	0.00	0.00	0.00	0.00	0.00

Continued on next page

Subgroups	Food Categories	Baby Cereal Consumption Groups					
		Baby cereal non-consumers		Non-rice baby cereal consumers		Rice baby cereal consumers	
4–6 months		Mean	SE	Mean	SE	Mean	SE
Poultry							
	Chicken, whole pieces	0.08	0.05	0.30	0.23	0.10	0.05
	Chicken patties, nuggets and tenders	0.00	0.00	0.06	0.06	0.02	0.02
	Turkey, duck, other poultry	0.00	0.00	0.00	0.00	0.04	0.04
Cured Meats/Poultry							
	Frankfurters	0.03	0.03	0.09	0.09	0.00	0.00
	Sausages	0.01	0.01	0.00	0.00	0.01	0.01
	Cold cuts and cured meats	0.00	0.00	0.02	0.02	0.00	0.00
Seafood							
	Fish	0.00	0.00	0.00	0.00	0.01	0.01
Other Desserts							
	Pudding	0.00	0.00	0.01	0.01	0.26	0.25
Sweet Bakery Products							
	Cookies and brownies	0.35	0.13	0.44	0.22	0.57	0.30
	Cakes and pies	0.00	0.00	0.01	0.01	0.12	0.11
Crackers							
	Saltine crackers	0.09	0.03	0.11	0.08	0.03	0.02
	Crackers, excludes saltines	0.06	0.03	0.38	0.30	0.07	0.03
Savory Snacks							
	Tortilla, corn, other chips	0.01	0.01	0.01	0.01	0.02	0.01
Candy							
	Candy not containing chocolate	0.00	0.00	0.14	0.12	0.00	0.00
Cooked Grains							
	Rice	0.31	0.19	0.88	0.83	0.29	0.24
	Pasta, noodles, cooked grains	0.12	0.09	0.00	0.00	0.04	0.04

Continued on next page

Subgroups	Food Categories	Baby Cereal Consumption Groups					
		Baby cereal non-consumers		Non-rice baby cereal consumers		Rice baby cereal consumers	
4–6 months		Mean	SE	Mean	SE	Mean	SE
Breads, Rolls, Tortillas							
	Yeast breads	0.17	0.10	0.49	0.39	0.06	0.03
	Tortillas	0.15	0.10	0.07	0.07	0.02	0.02
	Rolls and buns	0.02	0.01	0.35	0.25	0.03	0.02
Cooked Cereals							
	Grits and other cooked cereals	0.06	0.06	0.44	0.45	1.63	0.91
	Oatmeal	0.00	0.00	3.57	3.51	0.20	0.16
Ready-to-Eat Cereals							
	Ready-to-eat cereal, lower sugar (= < 21.2 g/100 g)	0.02	0.02	0.15	0.11	0.03	0.02
Quick Breads and Bread Products							
	Pancakes, waffles, French toast	0.01	0.01	0.00	0.00	0.00	0.00
	Biscuits, muffins, quick breads	0.00	0.00	0.00	0.00	0.01	0.01
Fats and Oils							
	Cream and cream substitutes	0.03	0.03	0.00	0.00	0.00	0.00
Condiments and Sauces							
	Dips, gravies, other sauces	0.00	0.00	0.03	0.02	0.00	0.00

Abbreviations: SE: Standard error. [a,b] Means with different superscripts indicate significant differences ($p < 0.01$) between baby cereal non-consumers and non-rice cereal consumers. [c,d] Means with different superscripts indicate significant differences ($p < 0.01$) between baby cereal non-consumers and baby rice cereal consumers. [e,f] Means with different superscripts indicate significant differences ($p < 0.01$) between non-rice cereal consumers and baby rice cereal consumers.

Supplemental Table 11. Introduction of foods for 7–11 months of age by baby cereal consumption groups.

Subgroups	Food Categories	Baby Cereal Consumption Groups					
		Baby cereal non-consumers		Non-rice baby cereal consumers		Rice baby cereal consumers	
7–11 months		Mean	SE	Mean	SE	Mean	SE
**Infant Formulas**							
	Formula, prepared from powder	410.06[c]	25.32	506.75	28.23	546.38[d]	25.33
	Formula, prepared from concentrate	26.40	5.83	41.85	10.93	46.12	8.01
	Formula, ready-to-feed	16.85[a]	6.44	55.58[b]	13.57	30.84	7.50
**Baby Foods**							
	Baby food: fruit	29.31[a,c]	3.24	71.50[b]	7.49	69.33[d]	6.60
	Baby food: meat and dinners	25.05[a,c]	3.64	49.61[b]	5.68	45.10[d]	5.42
	Baby food: vegetable	21.32[a,c]	2.38	54.59[b]	5.59	56.98[d]	4.56
	Baby food: snacks and sweets	7.41	1.39	11.21	1.91	9.70	1.66
	Baby food: yogurt	2.65	0.68	7.59	3.45	6.72	2.24
	Baby food: cereals	0.16[a,c]	0.16	40.05[b]	3.29	30.48[d]	2.24
**Baby Beverages**							
	Baby juice	24.48[a]	3.76	39.85[b]	4.85	38.50	4.99
	Baby water	8.06	2.00	7.60	2.04	7.54	2.12
**Milk**							
	Milk, whole	137.64[a,c]	12.86	50.87[b]	10.16	33.11[d]	7.89
	Milk, reduced fat	18.54[a,c]	3.46	3.03[b]	1.28	4.58[d]	1.69
	Milk, lowfat	8.64	4.16	1.74	1.57	0.19	0.14
	Milk, nonfat	0.25	0.25	0.00	0.00	0.00	0.00
**Yogurt**							
	Yogurt, regular	14.63[c]	2.78	6.92	1.44	4.46[d]	1.76
	Yogurt, Greek	0.66	0.29	0.00	0.00	0.00	0.00

Continued on next page

Subgroups	Food Categories	Baby Cereal Consumption Groups					
		Baby cereal non-consumers		Non-rice baby cereal consumers		Rice baby cereal consumers	
7–11 months		Mean	SE	Mean	SE	Mean	SE
**Dairy Drinks and Substitutes**							
	Milk substitutes	3.54	2.01	1.45	1.44	1.75	1.73
	Milk shakes and other dairy drinks	0.45	0.45	0.00[e]	0.00	0.28[f]	0.04
**Cheese**							
	Cheese	3.60[a]	0.54	1.20[b]	0.28	1.75	0.53
	Cottage/ricotta cheese	0.07	0.04	0.63	0.57	0.53	0.53
**Flavored Milk**							
	Flavored milk, whole	0.82	0.38	1.46	0.98	0.00	0.00
	Flavored milk, reduced fat	0.77	0.57	1.78	1.23	0.00	0.00
	Flavored milk, lowfat	0.22	0.22	0.00	0.00	0.60	0.60
**Plain Water**							
**Flavored or Enhanced Water**							
	Flavored or carbonated water	0.05	0.05	0.00	0.00	1.15	1.14
**100% Juice**							
	Apple juice	23.20[a]	3.52	10.04[b]	2.57	20.84	4.32
	Other fruit juice	18.71	3.48	13.03	5.30	10.98	3.52
	Citrus juice	5.68[a]	1.39	1.14[b]	0.50	2.68	0.96
	Vegetable juice	0.03	0.03	0.03	0.03	0.00	0.00
**Sweetened Beverages**							
	Fruit drinks	13.24[a]	2.76	3.95[b]	1.19	7.34	3.43
	Sport and energy drinks	6.88	2.45	0.51	0.25	6.67	2.42
	Smoothies and grain drinks	2.25	1.23	0.18	0.13	0.39	0.23
	Soft drinks	2.17	1.01	0.09	0.06	0.31	0.19

Continued on next page

Subgroups	Food Categories	Baby Cereal Consumption Groups					
		Baby cereal non-consumers		Non-rice baby cereal consumers		Rice baby cereal consumers	
7–11 months		Mean	SE	Mean	SE	Mean	SE
**Coffee and Tea**							
	Tea	3.28	1.16	2.47	1.20	1.87	0.90
	Coffee	0.01	0.01	0.21	0.21	0.00	0.00
**Diet Beverages**							
	Other diet drinks	0.07	0.05	1.34	1.32	0.00	0.00
	Diet soft drinks	0.00	0.00	0.00	0.00	0.35	0.27
**Mixed Dishes - Grain-based**							
	Pasta mixed dishes, excludes macaroni and cheese	16.89[a,c]	2.74	6.80[b]	1.80	5.65[d]	2.29
	Macaroni and cheese	10.75[a]	1.88	2.86[b]	0.90	4.51	1.68
	Rice mixed dishes	3.20	0.80	2.31	0.84	1.97	0.80
	Turnovers and other grain-based items	0.21	0.15	0.01	0.01	0.02	0.02
**Mixed Dishes - Soups**							
	Soups	21.10[c]	3.09	11.77	2.69	10.71[d]	2.03
**Mixed Dishes - Meat, Poultry, Seafood**							
	Meat mixed dishes	4.49[c]	1.11	3.37[e]	0.94	0.49[d,f]	0.25
	Poultry mixed dishes	2.82	0.93	1.59	0.67	2.12	0.68
**Mixed Dishes - Sandwiches (single code)**							
	Burgers (single code)	1.14	0.54	0.19	0.13	0.00	0.00
	Chicken/turkey sandwiches (single code)	0.28	0.24	0.00	0.00	0.00	0.00
**Mixed Dishes - Mexican**							
	Burritos and tacos	0.17	0.09	0.09	0.08	0.00	0.00

Continued on next page

Subgroups	Food Categories	Baby Cereal Consumption Groups					
		Baby cereal non-consumers		Non-rice baby cereal consumers		Rice baby cereal consumers	
7–11 months		Mean	SE	Mean	SE	Mean	SE
**Mixed Dishes - Pizza**							
	Pizza	1.42[a]	0.43	0.28[b]	0.09	0.27	0.19
**Mixed Dishes - Asian**							
	Fried rice and lo/chow mein	0.44	0.22	0.02	0.02	0.09	0.07
	Stir-fry and soy-based sauce mixtures	0.39	0.34	0.00	0.00	0.34	0.24
**Fruits**							
	Bananas	16.81[c]	2.21	13.03	2.09	6.99[d]	1.14
	Apples	13.30[a]	2.02	5.68[b]	1.63	6.31	1.80
	Peaches and nectarines	5.37[c]	1.38	3.11	1.03	0.99[d]	0.43
	Other fruits and fruit salads	5.00	1.15	4.13	1.16	3.68	1.17
	Berries	3.49[a]	1.13	0.24[b]	0.13	1.21	0.48
	Citrus fruits	2.26	0.60	1.77	1.15	1.05	0.42
	Melons	1.74	0.61	2.66	1.73	1.81	1.00
	Grapes	1.64[c]	0.50	0.55	0.21	0.19[d]	0.10
	Dried fruits	0.86	0.38	0.23	0.19	0.18	0.16
**Eggs**							
	Eggs and omelets	10.57[a,c]	1.43	3.21[b]	1.17	2.01[d]	0.55
**Plant-based Protein Foods**							
	Beans, peas, legumes	6.47	2.17	2.10	0.65	2.42	1.18
	Nuts and seeds	0.26[c]	0.09	0.37[e]	0.12	0.00[d,f]	0.00
	Processed soy products	0.72	0.50	0.01	0.01	0.30	0.23
**Poultry**							
	Chicken, whole pieces	3.98[a,c]	0.80	0.85[b]	0.26	1.48[d]	0.40
	Chicken patties, nuggets and tenders	2.78[a,c]	0.54	0.53[b]	0.24	0.73[d]	0.26

Continued on next page

Subgroups	Food Categories	Baby Cereal Consumption Groups					
		Baby cereal non-consumers		Non-rice baby cereal consumers		Rice baby cereal consumers	
7–11 months		Mean	SE	Mean	SE	Mean	SE
**Cured Meats/Poultry**							
	Frankfurters	2.50	0.79	0.46	0.29	0.63	0.30
	Cold cuts and cured meats	1.71	0.46	1.01	0.41	0.61	0.37
	Sausages	1.62	0.72	0.15	0.09	0.24	0.17
	Bacon	0.06	0.03	0.12	0.11	0.00	0.00
**Meats**							
	Ground beef	0.73	0.37	0.14	0.12	0.59	0.38
	Beef, excludes ground	0.36	0.15	0.64	0.31	0.13	0.10
	Pork	0.24	0.09	0.23	0.14	0.02	0.02
	Lamb, goat, game	0.18	0.12	0.00	0.00	0.00	0.00
	Liver and organ meats	0.05	0.05	0.00	0.00	0.00	0.00
**Seafood**							
	Fish	0.46	0.19	0.08	0.05	0.22	0.18
	Shellfish	0.05	0.03	0.00	0.00	0.09	0.08
**Vegetables, excluding Potatoes**							
	Other vegetables and combinations	6.99	1.71	3.31	1.07	2.92	1.20
	Other starchy vegetables	2.74	0.99	0.69	0.34	0.73	0.38
	String beans	2.29	0.68	1.44	0.46	1.09	0.47
	Dark green vegetables, excludes lettuce	2.06	0.57	0.50	0.17	1.14	0.66
	Other red and orange vegetables	2.05	0.86	3.24	1.04	0.94	0.45
	Carrots	1.96	0.51	1.59	0.51	2.33	1.28
	Tomatoes	1.85	1.29	0.01	0.01	0.18	0.12

Continued on next page

Subgroups	Food Categories	Baby Cereal Consumption Groups					
		Baby cereal non-consumers		Non-rice baby cereal consumers		Rice baby cereal consumers	
7–11 months		Mean	SE	Mean	SE	Mean	SE
**Vegetables, excluding Potatoes**							
	Corn	1.55[a]	0.47	0.00[b]	0.00	0.29	0.14
	Vegetable mixed dishes	0.49	0.23	0.00	0.00	0.00	0.00
	Onions	0.02	0.01	0.00	0.00	0.01	0.01
**White Potatoes**							
	Mashed potatoes and white potato mixtures	6.12	1.31	3.31	1.03	4.94	1.12
	French fries and other fried white potatoes	2.99[a,c]	0.65	0.77[b]	0.26	0.65[d]	0.24
	White potatoes, baked or boiled	0.76	0.31	0.75	0.36	0.50	0.36
**Cooked Cereals**							
	Oatmeal	6.06[a,c]	1.20	1.29[b]	0.70	0.64[d]	0.29
	Grits and other cooked cereals	4.13	1.97	0.98	0.71	1.51	0.69
**Breads, Rolls, Tortillas**							
	Yeast breads	5.28[a,c]	0.66	1.74[b]	0.44	1.99[d]	0.54
	Tortillas	1.33[a]	0.26	0.40[b]	0.14	0.69	0.29
	Rolls and buns	0.67	0.18	0.59	0.49	0.44	0.15
	Bagels and English muffins	0.38	0.18	0.20	0.19	0.03	0.03
**Cooked Grains**							
	Rice	3.12	0.90	3.99	1.03	3.17	1.41
	Pasta, noodles, cooked grains	1.08	0.37	0.77	0.34	0.41	0.17

Continued on next page

Subgroups	Food Categories	Baby Cereal Consumption Groups					
		Baby cereal non-consumers		Non-rice baby cereal consumers		Rice baby cereal consumers	
**7–11 months**		**Mean**	**SE**	**Mean**	**SE**	**Mean**	**SE**
**Quick Breads and Bread Products**							
	Pancakes, waffles, French toast	2.54[a,c]	0.64	0.61[b]	0.22	0.44[d]	0.23
	Biscuits, muffins, quick breads	1.29	0.37	1.08	0.89	0.65	0.38
**Ready-to-Eat Cereals**							
	Ready-to-eat cereal, lower sugar (= < 21.2 g/100 g)	1.85	0.32	1.17	0.24	1.11	0.32
	Ready-to-eat cereal, higher sugar (> 21.2 g/100 g)	1.22[a,c]	0.21	0.21[b]	0.11	0.30[d]	0.13
**Sweet Bakery Products**							
	Cookies and brownies	4.45[a,c]	0.45	1.44[b]	0.28	1.79[d]	0.28
	Cakes and pies	1.38	0.60	0.32	0.29	0.20	0.13
	Doughnuts, sweet rolls, pastries	1.03	0.38	0.44	0.21	0.14	0.06
**Other Desserts**							
	Gelatins, ices, sorbets	1.56	0.49	0.55	0.31	1.06	0.49
	Ice cream and frozen dairy desserts	1.39[a]	0.31	0.15[b]	0.08	1.26	0.48
	Pudding	1.28	0.34	0.60	0.53	0.88	0.59
**Crackers**							
	Crackers, excludes saltines	1.62	0.29	1.08	0.23	1.68	0.34
	Saltine crackers	0.57[a]	0.10	0.12[b]	0.05	0.48	0.15

Continued on next page

Subgroups	Food Categories	Baby Cereal Consumption Groups					
		Baby cereal non-consumers		Non-rice baby cereal consumers		Rice baby cereal consumers	
7–11 months		Mean	SE	Mean	SE	Mean	SE
**Savory Snacks**							
	Tortilla, corn, other chips	1.12	0.29	0.60	0.18	0.63	0.18
	Pretzels/snack mix	0.24	0.11	0.15	0.09	0.15	0.08
	Popcorn	0.05	0.03	0.01	0.01	0.03	0.03
**Snack/Meal Bars**							
	Cereal bars	0.71	0.37	0.21	0.16	0.01	0.01
	Nutrition bars	0.05	0.05	0.00	0.00	0.00	0.00
**Candy**							
	Candy not containing chocolate	0.63	0.29	0.42	0.20	0.24	0.16
	Candy containing chocolate	0.09	0.06	0.01	0.01	0.00	0.00
**Condiments and Sauces**							
	Tomato-based condiments	0.14	0.09	0.00[e]	0.00	0.02[f]	0.00
	Mustard and other condiments	0.13	0.11	0.00	0.00	0.00	0.00
**Fats and Oils**							
	Margarine	0.29[a]	0.08	0.04[b]	0.03	0.11	0.05
	Butter and animal fats	0.20	0.07	0.05	0.02	0.05	0.03
	Cream cheese, sour cream, whipped cream	0.05	0.03	0.00	0.00	0.00	0.00
	Mayonnaise	0.04	0.02	0.01	0.00	0.03	0.02
	Salad dressings and vegetable oils	0.00	0.00	0.00	0.00	0.02	0.02
**Sugars**							
	Jams, syrups, toppings	0.36	0.10	0.10	0.04	0.21	0.10
	Sugars and honey	0.09	0.05	0.09	0.07	0.03	0.02

Continued on next page

Subgroups	Food Categories	Baby Cereal Consumption Groups					
		Baby cereal non-consumers		Non-rice baby cereal consumers		Rice baby cereal consumers	
7–11 months		Mean	SE	Mean	SE	Mean	SE
**Other**							
	Not included in a food category	0.08	0.04	0.03	0.03	0.00	0.00

Abbreviations: SE: Standard error. [a,b] Means with different superscripts indicate significant differences ($p < 0.01$) between baby cereal non-consumers and non-rice cereal consumers. [c,d] Means with different superscripts indicate significant differences ($p < 0.01$) between baby cereal non-consumers and baby rice cereal consumers. [c,f] Means with different superscripts indicate significant differences ($p < 0.01$) between non-rice cereal consumers and baby rice cereal consumers.

Supplemental Table 12. Introduction of foods for 12–23 months of age by baby cereal consumption groups.

Subgroups	Food Categories	Baby Cereal Consumption Groups					
		Baby cereal non-consumers		Non-rice baby cereal consumers		Rice baby cereal consumers	
12–23 months		Mean	SE	Mean	SE	Mean	SE
Milk							
	Milk, whole	354.06	10.56	355.69	37.37	415.51	62.32
	Milk, reduced fat	104.41^{a,c}	8.60	38.11^{b}	12.05	15.89^{d}	8.45
	Milk, lowfat	9.69^{a}	1.74	0.99^{b}	1.00	21.34	17.22
	Milk, nonfat	7.50^{a,c}	2.29	0.12^{b}	0.12	0.00^{d}	0.00
Yogurt							
	Yogurt, regular	20.23	1.72	22.35	7.80	39.46	14.55
	Yogurt, Greek	1.91^{a}	0.72	0.00^{b}	0.00	0.68	0.68
Flavored milk							
	Flavored milk, whole	12.21	2.73	6.80	6.81	36.70	32.00
	Flavored milk, reduced fat	3.92^{c}	0.99	2.05	1.74	0.00^{d}	0.00
	Flavored milk, lowfat	2.08	1.16	0.00	0.00	0.00	0.00
	Flavored milk, nonfat	0.70	0.36	0.00	0.00	0.00	0.00

Continued on next page

Subgroups	Food Categories	Baby Cereal Consumption Groups					
		Baby cereal non-consumers		Non-rice baby cereal consumers		Rice baby cereal consumers	
12–23 months		Mean	SE	Mean	SE	Mean	SE
Dairy drinks and substitutes							
	Milk substitutes	15.24[c]	2.71	46.69	24.65	0.00[d]	0.00
	Milk shakes and other dairy drinks	1.26	0.53	0.09	0.09	0.00	0.00
Cheese							
	Cheese	9.36[a,c]	0.66	5.54[b]	1.31	4.70[d]	1.10
	Cottage/ricotta cheese	1.86[c]	0.64	0.54	0.43	0.00[d]	0.00
100 % Juice							
	Apple juice	73.82	5.11	50.12	11.68	79.42	19.56
	Other fruit juice	56.56[c]	5.20	48.17	13.51	12.10[d]	7.27
	Citrus juice	24.01	2.07	16.14	12.48	34.45	16.83
	Vegetable juice	1.28	0.66	0.12	0.12	0.00	0.00
Sweetened Beverages							
	Fruit drinks	73.12	5.63	46.73	24.54	50.44	23.82
	Soft drinks	16.82	3.06	13.35	10.40	6.87	3.18
	Sport and energy drinks	13.33	3.88	3.58	2.46	3.17	1.89
	Smoothies and grain drinks	4.26[a]	1.09	0.00[b]	0.00	5.85	4.59
Coffee and Tea							
	Tea	15.94[c]	3.42	7.09	3.73	0.00[d]	0.00
	Coffee	0.47	0.23	0.00	0.00	1.52	1.50
Diet Beverages							
	Other diet drinks	3.64[c]	1.26	5.94	5.23	0.00[d]	0.00
	Diet soft drinks	1.21	0.49	0.22	0.21	1.69	1.26
Plain Water							
	Tap water	115.10[c]	7.46	100.86	23.26	51.45[d]	14.03
	Bottled water	67.17	5.59	72.84	22.26	59.05	16.99

Continued on next page

Subgroups	Food Categories	Baby Cereal Consumption Groups					
		Baby cereal non-consumers		Non-rice baby cereal consumers		Rice baby cereal consumers	
12–23 months		Mean	SE	Mean	SE	Mean	SE
Flavored or enhanced water							
	Enhanced or fortified water	1.32	1.00	0.00	0.00	2.17	2.12
	Flavored or carbonated water	0.94	0.41	0.00	0.00	0.00	0.00
Mixed Dishes - Grain-based							
	Pasta mixed dishes, excludes macaroni and cheese	23.70	2.06	18.36	5.43	23.74	9.49
	Macaroni and cheese	16.45[a,c]	1.66	5.80[b]	2.52	3.77[d]	2.31
	Rice mixed dishes	6.66	0.75	2.71	1.50	6.97	2.82
	Turnovers and other grain-based items	0.76[c]	0.29	0.99	0.99	0.00[d]	0.00
	Processed soy products	0.74	0.28	0.00	0.00	0.00	0.00
Mixed Dishes - Soups							
	Soups	31.98	3.91	23.18	7.86	41.32	12.02
Mixed Dishes - Meat, Poultry, Seafood							
	Meat mixed dishes	9.25	1.31	9.33	2.81	4.63	4.34
	Poultry mixed dishes	7.16	1.18	20.70	8.22	8.65	5.34
	Seafood mixed dishes	1.80[a]	0.54	0.03[b]	0.03	9.04	9.31
Mixed Dishes - Pizza							
	Pizza	8.29[a,c]	0.73	2.48[b]	1.40	1.92[d]	1.12
Mixed Dishes - Mexican							
	Other Mexican mixed dishes	3.77[a,c]	0.76	0.94[b]	0.64	0.26[d]	0.25
	Burritos and tacos	2.89	0.62	1.17	0.82	2.19	2.18
	Nachos	0.06	0.03	0.00	0.00	0.00	0.00

Continued on next page

Subgroups	Food Categories	Baby Cereal Consumption Groups					
		Baby cereal non-consumers		Non-rice baby cereal consumers		Rice baby cereal consumers	
**12–23 months**		**Mean**	**SE**	**Mean**	**SE**	**Mean**	**SE**
Mixed Dishes - Sandwiches (single code)							
	Burgers (single code)	2.42[a,c]	0.43	0.49[b]	0.38	0.00[d]	0.00
	Frankfurter sandwiches (single code)	1.50[a,c]	0.43	0.00[b]	0.00	0.00[d]	0.00
	Other sandwiches (single code)	0.73	0.34	0.00	0.00	0.00	0.00
	Peanut butter and jelly sanwiches (single code)	0.58[a,c]	0.16	0.00[b]	0.00	0.00[d]	0.00
	Chicken/turkey sandwiches (single code)	0.49	0.22	0.00	0.00	0.00	0.00
	Egg/breakfast sandwiches (single code)	0.34[a,c]	0.12	0.00[b]	0.00	0.00[d]	0.00
	Cheese sandwiches (single code)	0.33	0.19	0.00	0.00	0.00	0.00
Mixed Dishes - Asian							
	Fried rice and lo/chow mein	1.73[a]	0.56	0.10[b]	0.10	0.66	0.67
	Stir-fry and soy-based sauce mixtures	0.99[c]	0.31	0.83	0.54	0.00[d]	0.00
	Egg rolls, dumplings, sushi	0.16	0.06	0.17	0.17	1.54	1.27

Continued on next page

Subgroups	Food Categories	Baby Cereal Consumption Groups					
		Baby cereal non-consumers		Non-rice baby cereal consumers		Rice baby cereal consumers	
12–23 months		Mean	SE	Mean	SE	Mean	SE
Fruits							
	Bananas	23.97	1.46	16.77	4.83	15.96	5.75
	Apples	20.85	1.60	34.55	8.74	22.82	6.40
	Other fruits and fruit salads	11.22	1.24	12.50	3.83	10.72	3.90
	Grapes	8.11	0.98	4.90	2.35	6.69	4.66
	Citrus fruits	8.00	1.05	7.82	3.14	7.62	3.84
	Berries	6.73	0.77	4.11	2.80	4.34	3.38
	Melons	5.95	0.95	7.71	6.30	5.71	2.46
	Peaches and nectarines	4.31	0.59	8.42	4.61	1.66	1.04
	Dried fruits	1.34[c]	0.28	0.78	0.69	0.33[d]	0.24
Cooked Cereals							
	Oatmeal	15.44	1.71	8.28	4.42	8.56	4.09
	Grits and other cooked cereals	4.55	0.76	4.06	1.95	3.42	3.15
Breads, Rolls, Tortillas							
	Yeast breads	11.08	0.70	13.84	3.86	5.48	2.06
	Rolls and buns	2.30[a,c]	0.29	0.73[b]	0.53	0.00[d]	0.00
	Tortillas	2.08	0.20	2.20	1.20	2.47	0.87
	Bagels and English muffins	1.24[c]	0.27	0.38	0.37	0.00[d]	0.00
Cooked Grains							
	Rice	7.58	0.71	10.32	3.51	5.59	2.39
	Pasta, noodles, cooked grains	4.93[a,c]	0.87	0.20[b]	0.20	0.67[d]	0.47
Ready-to-Eat Cereals							
	Ready-to-eat cereal, higher sugar (> 21.2 g/100 g)	4.61[c]	0.32	2.92	1.24	1.33[d]	0.60
	Ready-to-eat cereal, lower sugar (= <21.2 g/100 g)	4.05	0.30	3.49	1.23	2.56	1.08

Continued on next page

Subgroups	Food Categories	Baby Cereal Consumption Groups					
		Baby cereal non-consumers		Non-rice baby cereal consumers		Rice baby cereal consumers	
12–23 months		Mean	SE	Mean	SE	Mean	SE
Quick Breads and Bread Products							
	Pancakes, waffles, French toast	5.05[a]	0.59	0.75[b]	0.46	3.92	2.26
	Biscuits, muffins, quick breads	1.97[c]	0.31	1.37	0.81	0.00[d]	0.00
Poultry							
	Chicken patties, nuggets and tenders	8.95	0.84	4.03	2.15	5.98	2.91
	Chicken, whole pieces	8.35	0.68	9.71	3.42	3.45	1.95
	Turkey, duck, other poultry	0.87[a]	0.18	0.00[b]	0.00	1.20	1.11
Eggs							
	Eggs and omelets	17.30[a]	1.18	6.60[b]	2.22	11.58	3.52
Cured Meats/Poultry							
	Frankfurters	4.85[a,c]	0.57	1.20[b]	1.04	1.07[d]	0.78
	Cold cuts and cured meats	4.23	0.40	3.02	1.44	2.95	1.38
	Sausages	2.92[a,c]	0.45	0.60[b]	0.38	0.07[d]	0.06
	Bacon	0.45[a,c]	0.08	0.00[b]	0.00	0.01[d]	0.01
Plant-based Protein Foods							
	Beans, peas, legumes	6.70	0.75	3.26	1.99	5.09	2.26
	Nuts and seeds	1.78[c]	0.24	2.01	0.74	0.35[d]	0.17
	Processed soy products	0.74[a,c]	0.28	0.00[b]	0.00	0.00[d]	0.00

Continued on next page

Subgroups	Food Categories	Baby Cereal Consumption Groups					
		Baby cereal non-consumers		Non-rice baby cereal consumers		Rice baby cereal consumers	
12–23 months		Mean	SE	Mean	SE	Mean	SE
Meats							
	Beef, excludes ground	2.15	0.28	1.24	0.65	3.55	2.69
	Ground beef	1.57	0.26	1.15	1.15	0.68	0.67
	Pork	1.45[c]	0.28	0.46	0.46	0.15[d]	0.15
	Lamb, goat, game	0.06	0.04	0.00	0.00	0.14	0.14
	Liver and organ meats	0.03	0.03	0.00	0.00	0.00	0.00
Seafood							
	Fish	1.81	0.33	0.66	0.50	2.01	1.79
	Shellfish	0.18	0.07	0.00	0.00	0.00	0.00
Infant Formulas							
	Formula, prepared from powder	13.21	2.67	72.18	24.34	132.08	46.62
Baby Foods							
	Baby food: meat and dinners	6.04[a]	1.11	37.33[b]	10.42	18.57	8.08
	Baby food: fruit	3.87[a]	1.04	44.75[b]	12.40	18.07	5.61
	Baby food: vegetable	2.38[a]	0.52	38.68[b]	11.19	8.44	3.10
	Baby food: yogurt	2.33	0.85	2.34	1.82	3.33	3.33
	Baby food: snacks and sweets	1.58	0.22	9.89	3.53	12.83	7.49
	Baby food: cereals	0.00[a,c]	0.00	43.74[b]	5.06	25.58[d]	5.35
Baby Beverages							
	Baby juice	10.90	1.93	33.49	13.60	14.73	7.27
Sweet Bakery Products							
	Cookies and brownies	9.24	0.59	8.64	1.70	9.68	2.59
	Cakes and pies	3.42[a,c]	0.50	0.94[b]	0.76	0.48[d]	0.48
	Doughnuts, sweet rolls, pastries	2.89	0.32	2.29	1.10	1.24	0.79

Continued on next page

Subgroups	Food Categories	Baby Cereal Consumption Groups					
		Baby cereal non-consumers		Non-rice baby cereal consumers		Rice baby cereal consumers	
**12–23 months**		**Mean**	**SE**	**Mean**	**SE**	**Mean**	**SE**
Other Desserts							
	Ice cream and frozen dairy desserts	5.99[a,c]	0.75	1.29[b]	0.54	0.39[d]	0.39
	Gelatins, ices, sorbets	4.80[c]	0.54	1.90	1.13	1.48[d]	1.00
	Pudding	2.16[a]	0.41	0.46[b]	0.34	1.52	1.49
Crackers							
	Crackers, excludes saltines	6.12[a]	0.40	3.07[b]	1.06	3.56	1.75
	Saltine crackers	0.74	0.11	0.88	0.31	0.53	0.26
Savory Snacks							
	Tortilla, corn, other chips	2.99[c]	0.26	1.89	0.93	0.47[d]	0.25
	Potato chips	1.54[c]	0.12	1.23	0.58	0.60[d]	0.27
	Popcorn	0.41	0.06	0.26	0.17	0.15	0.12
Candy							
	Candy not containing chocolate	3.60	0.35	1.86	0.89	2.99	1.73
	Candy containing chocolate	1.14	0.35	0.37	0.23	0.66	0.33
Snack/Meal Bars							
	Cereal bars	1.59	0.26	1.56	1.25	0.48	0.48
	Nutrition bars	0.06	0.04	0.00	0.00	0.00	0.00

Continued on next page

Subgroups	Food Categories	Baby Cereal Consumption Groups					
		Baby cereal non-consumers		Non-rice baby cereal consumers		Rice baby cereal consumers	
12–23 months		Mean	SE	Mean	SE	Mean	SE
Vegetables, excluding Potatoes							
	Other vegetables and combinations	5.74[c]	0.72	4.98	2.67	0.31[d]	0.30
	String beans	4.87[c]	0.69	4.43	1.46	0.68[d]	0.50
	Corn	3.77[a,c]	0.54	0.75[b]	0.47	0.00[d]	0.00
	Dark green vegetables, excludes lettuce	2.98	0.53	1.43	0.74	1.18	0.62
	Carrots	2.78	0.34	2.35	1.31	1.42	0.84
	Tomatoes	2.60[a,c]	0.41	0.31[b]	0.20	0.38[d]	0.38
	Other starchy vegetables	2.25[c]	0.40	3.22	1.27	0.35[d]	0.25
	Vegetable mixed dishes	2.19[a]	0.38	0.45[b]	0.40	2.29	2.09
	Other red and orange vegetables	1.59	0.47	2.17	1.38	4.44	3.01
	Lettuce and lettuce salads	0.59	0.09	0.31	0.17	0.15	0.15
	Onions	0.08[c]	0.02	0.07	0.07	0.00[d]	0.00
White Potatoes							
	French fries and other fried white potatoes	7.74[a,c]	0.59	1.68[b]	0.91	1.79[d]	1.07
	Mashed potatoes and white potato mixtures	7.44[c]	0.83	13.28	7.65	1.21[d]	1.26
	White potatoes, baked or boiled	2.98[c]	0.48	1.66	1.14	0.56[d]	0.34

Continued on next page

Subgroups	Food Categories	Baby Cereal Consumption Groups					
		Baby cereal non-consumers		Non-rice baby cereal consumers		Rice baby cereal consumers	
**12–23 months**		**Mean**	**SE**	**Mean**	**SE**	**Mean**	**SE**
Condiments and Sauces							
	Dips, gravies, other sauces	1.79[a,c]	0.41	0.03[b]	0.03	0.03[d]	0.03
	Pasta sauces, tomato-based	1.38[c]	0.36	1.75	1.73	0.00[d]	0.00
	Tomato-based condiments	0.97[c]	0.12	0.89	0.41	0.27[d]	0.15
	Olives, pickles, pickled vegetables	0.68[a,c]	0.15	0.00[b]	0.00	0.14[d]	0.14
	Mustard and other condiments	0.16[a]	0.05	0.00[b]	0.00	0.10	0.07
	Soy-based condiments	0.05	0.02	0.00	0.00	0.02	0.02
Sugars							
	Jams, syrups, toppings	2.08[c]	0.20	1.12	0.51	0.59[d]	0.33
	Sugars and honey	0.40	0.06	0.22	0.13	0.69	0.32
Fats and Oils							
	Butter and animal fats	0.43	0.05	0.22	0.10	0.28	0.15
	Salad dressings and vegetable oils	0.40[c]	0.07	0.27	0.22	0.00[d]	0.00
	Margarine	0.36[c]	0.05	0.16	0.07	0.03[d]	0.03
	Cream cheese, sour cream, whipped cream	0.27	0.07	0.08	0.08	0.17	0.13
	Mayonnaise	0.20	0.04	0.11	0.08	0.20	0.18
Other							
	Not included in a food category	0.34[a]	0.11	0.04[b]	0.03	1.71	1.61

Continued on next page

Subgroups	Food Categories	Baby Cereal Consumption Groups					
		Baby cereal non-consumers		Non-rice baby cereal consumers		Rice baby cereal consumers	
12–23 months		Mean	SE	Mean	SE	Mean	SE
Protein and Nutritional Powders							
	Protein and nutritional powders	0.14	0.08	0.00	0.00	0.00	0.00

Abbreviations: SE: Standard error. [a,b] Means with different superscripts indicate significant differences ($p < 0.01$) between baby cereal non-consumers and non-rice cereal consumers. [c,d] Means with different superscripts indicate significant differences ($p < 0.01$) between baby cereal non-consumers and baby rice cereal consumers. [e,f] Means with different superscripts indicate significant differences ($p < 0.01$) between non-rice cereal consumers and baby rice cereal consumers.

AIMS Press

© 2020 the Author(s), licensee AIMS Press. This is an open access article distributed under the terms of the Creative Commons Attribution License (http://creativecommons.org/licenses/by/4.0)
